# Cranial anatomy of the Middle Eocene Egyptian crocodylian *Herugavialis cairensis* provides new insights into gavialoid diversity in the Mediterranean region

**DOI:** 10.1098/rsos.261173

**Published:** 2026-10-07

**Authors:** Paul M. J. Burke, Stephan N. F. Spiekman, Belal S. Salem, Sara Saber, Hesham M. Sallam, Philip D. Mannion

**Affiliations:** ^1^ Department of Earth Sciences, University College London, London, UK; ^2^ Department of Palaeobiology, Naturhistoriska riksmuseet, Stockholm, Sweden; ^3^ Staatliches Museum für Naturkunde Stuttgart, Stuttgart, Germany; ^4^ Department of Paleontology, University of Hohenheim, Stuttgart, Germany; ^5^ Mansoura University Vertebrate Paleontology Center (MUVP), Faculty of Science, Mansoura University, Mansoura 35516, Egypt; ^6^ Department of Biological Sciences, Ohio University, Athens, Ohio 45701, USA; ^7^ Department of Geology, Faculty of Science, Benha University, Benha 13518, Egypt; ^8^ Department of Geology, Faculty of Science, Assiut University, Assuit 7515, Egypt

**Keywords:** Gavialoidea, Mediterranean, Crocodylia, *Tomistoma*, neuroanatomy, phylogeny

## Abstract

Gavialoidea is presently composed of only two extant crocodylian species, the Indian gharial *Gavialis gangeticus,* and the false gharial *Tomistoma schlegelii*, both from southeast Asia. However, Gavialoidea displays a rich and near-cosmopolitan diversity in the fossil record, with over 40 species currently recognized. Several of these gavialoids are recovered from Cenozoic deposits in the Mediterranean region, with many assigned historically to the genus *Tomistoma*. However, recent phylogenetic analyses do not typically recover these species as closely related to the false gharial, with several fossil species in need of taxonomic revision. Here, we present a detailed re-evaluation of one of these species, *Tomistoma cairense* from the Middle Eocene Mokattam Formation of Egypt, based on the holotype skull and several referred specimens. Supplemented by endocranial anatomy revealed via computed tomography (CT), we identify numerous anatomical differences with *T. schlegelii* and other gavialoids, including the approximately contemporaneous Egyptian species, *Eogavialis africanum* and *Paratomistoma courti*. These differences are reinforced through phylogenetic analysis under maximum parsimony, in which *T. cairense* is recovered as a gavialoid that does not cluster with *T. schlegelii* or Mediterranean taxa. We erect a new genus name for *T. cairense*, with the resultant new combination of *Herugavialis cairensis* diagnosed by several characteristics, including telescoped orbits. An apparent decline in Mediterranean gavialoid diversity during the Late Eocene–Oligocene is potentially driven by a reduction in habitat, as a result of global cooling and declining eustatic sea level. Warming in the Miocene might have facilitated a subsequent rebound in gavialoid diversity, prior to the extirpation of crocodylians in the Mediterranean region in the Late Miocene, which might have been caused by the Messinian Salinity Crisis. The taxonomic revision of additional Mediterranean gavialoid species should facilitate greater insight into their evolutionary history and demise.

## 1. Introduction

The gavialid crocodylian, *Tomistoma* is represented by one extant species, *Tomistoma schlegelii*, the false gharial, which is restricted to freshwater environments in southeast Asia and classified as Endangered based on the IUCN Red List [1,2]. By contrast, a large number of Cenozoic fossil species have historically been assigned to this genus, such that the fossil record implies a greater diversity and broader past distribution of *Tomistoma*, extending its stratigraphic range back to the Eocene and its geographic range to Africa and Europe [3]. It has long been recognized that at least some of these extinct species are unlikely to belong to *Tomistoma*, but, until recently, phylogenetic analyses of fossil crocodylians have recovered several species assigned to the genus as close relatives of *T. schlegelii* (e.g. [4–7]). This began to change with the study of Iijima & Kobayashi [8], whose phylogenetic analyses recovered none of these fossil species as closely related to *T. schlegelii*. Most subsequent studies have supported this, with, at most, a single species (the Miocene European taxon, *Tomistoma lusitanica*) sometimes recovered as closely related to *T. schlegelii* (e.g. [9–13]). As such, the genus-level taxonomic classification of many of these fossil species requires re-evaluation. Thus far, the Pleistocene East Asian species, *Tomistoma taiwanicus* [14], the Miocene North African species, *Tomistoma dowsoni* [15] and the Eocene East Asian species, *Tomistoma petrolica* [16], have formally been assigned to other genera, that is, *Toyotamaphimeia taiwanicus*, *Sutekhsuchus dowsoni* and *Maomigosuchus petrolica*, respectively [17–19], leaving several species in open nomenclature and in need of taxonomic revision [3,7].

Most of these remaining species come from the Mediterranean region [7,20]. One such species is ‘*Tomistoma*’ *cairense*, from the Middle Eocene of Egypt, which is one of the stratigraphically oldest taxa to be assigned to the genus. Known from several specimens, including the fairly complete holotype skull Staatliches Museum für Naturkunde Stuttgart (SMNS) 10575, Müller [21] assigned this species to *Tomistoma* based on several anatomical similarities he noted with *T. schlegelii*. Recent phylogenetic analyses have consistently recovered this species as a gavialid gavialoid that is not closely related to *T. schlegelii* (e.g. [10,13,22,23]), indicating that it warrants reclassification. However, the phylogenetic position of ‘*Tomistoma*’ *cairense* within Gavialoidea is unstable (e.g. [9]), and its taxonomic identification is potentially further complicated in that another gavialoid genus, *Paratomistoma courti* [24], is already recognized from approximately contemporaneous deposits in Egypt. As such, it is unclear if ‘*Tomistoma*’ *cairense* is referable to an existing genus of extinct gavialoid. Finally, the anatomy of ‘*Tomistoma*’ *cairense* has not been described since the erection of this species a century ago.

Here, we present a redescription of the holotype and referred remains of ‘*Tomistoma*’ *cairense*, utilizing computed tomography (CT) scans to garner information on its internal cranial anatomy. We use this new information to re-evaluate the systematics of ‘*Tomistoma*’ *cairense*. We make anatomical comparisons with other gavialoids, especially species from the Eocene of Egypt, and additionally present new anatomical data for *P. courti*. Finally, we discuss the diversity of extinct gavialoids in the Mediterranean region.

## 2. Material and methods

### 2.1. Specimen scanning and reconstructions

The holotype skull of *T. cairense* (SMNS 10575) was characterized at Carl Zeiss IQS Deutschland with X-ray micro-CT using a Zeiss Xradia Ultra. The endocranial anatomy of SMNS 10575 was subsequently segmented in Avizo v. 9.7 (FEI Visualization Science Group; https://www.thermofisher.com). The holotype skull of *P. courti* (Cairo Geological Museum (CGM) 42188) was surface scanned using an Artec Leo 3D scanner provided by Mansoura University Vertebrate Paleontology (MUVP) Center. This was then converted to a volume rendering and partially segmented in Avizo v.9.7 to reconstruct the encephalic endocast. The reconstructions of both specimens were subsequently smoothed in Blender (Stichting Blender Foundation, Amsterdam), and formatted in Inkscape (Inkscape Project, (2020). Inkscape Retrived from https://inkscape.org). Scan data for both specimens are available on MorphoSource (https://www.morphosource.org/projects/000868536?locale%3Den).

### 2.2. Phylogenetic approach

We used the phylogenetic data matrix published by Boerman *et al.* [25], which is the most recent iteration of the crocodylian-focused matrix originally published by Rio & Mannion [10]. This matrix already includes an operational taxonomic unit (OTU) for ‘*Tomistoma*’ *cairense*, to which we rescored 49 characters (see supplementary material for a list of character score changes). Rio & Mannion [10] included scores for the surangular and angular; however, neither of these elements is preserved in the type or referred materials, and as such these elements are not scored herein. Our revised OTU for ‘*Tomistoma*’ *cairense* is scored for a total of 126 of 331 characters. We also revised the scores of 10 characters for the existing *P. courti* OTU based on our study of CGM 42188 (see supplementary material for a list of character score changes). Finally, we incorporated the Late Miocene–Pliocene Ugandan species ‘*Tomistoma*’ *coppensi* [26], based on photographs of the type specimen provided by C. A. Brochu. The resultant data matrix consists of 152 OTUs scored for 331 morphological characters, representing an extensive sample of crocodylian taxa. The data matrix is provided as a TNT file in the supplementary material.

The data matrix was analysed under maximum parsimony in TNT v. 1.6 [27], with *Bernissartia fagesii* set as the outgroup taxon, and quantitative characters treated as continuous data (see [10]). The matrix was analysed under both equal weighting and extended implied weighting methodologies. Extended implied weighting downweights homoplastic characters, whereby a weight is calculated for each character depending on fit to a tree [28]. The *k* value (the concavity constant) was set to 12, following Rio & Mannion [10]. For all analyses, we used the ‘Stabilize Consensus' option in ‘New Technology Search’, with all algorithms for each analysis enabled, and the consensus tree stabilized five times with a factor of 75. This was subsequently followed by a traditional search using trees from RAM. Following previous iterations of this data matrix, 37 characters were treated as ordered (characters 17, 37, 47, 48, 58, 65, 72, 75, 78, 81, 87, 88, 102, 109, 110, 137, 142, 151, 162, 175, 181, 188, 210, 214, 216, 220, 221, 222, 224, 235, 243, 284, 293, 297, 308, 323 and 324). After each analysis, bootstrap and jackknife support values were calculated as frequency differences in TNT as per the methodology followed in Boerman *et al.* [25]. The consensus tree from the extended implied weighting analysis was subsequently used to time scale the tree using stratigraphic ranges in the R package ‘strap’ [29], using the stratigraphic ages of the first and last known appearances of the OTUs listed in Rio & Mannion [10], supplemented by The Paleobiology Database (https://paleobiodb.org/) and the recent literature.

## 3. Systematic palaeontology

Eusuchia Huxley, 1875 [30],

Crocodylia Gmelin, 1788 [31],
Gavialoidea Hay, 1930 [32],
*Herugavialis* gen. nov,
ZooBank LSID: zoobank.org:pub:0A77BBDD-67CA-49AA-80A4-AACA88938A20,
*Herugavialis cairensis* n. comb. Müller, 1927 [21],
Tomistoma cairense (Müller, 1927 [21]).
(Figures 1–8)

**Figure 1.**
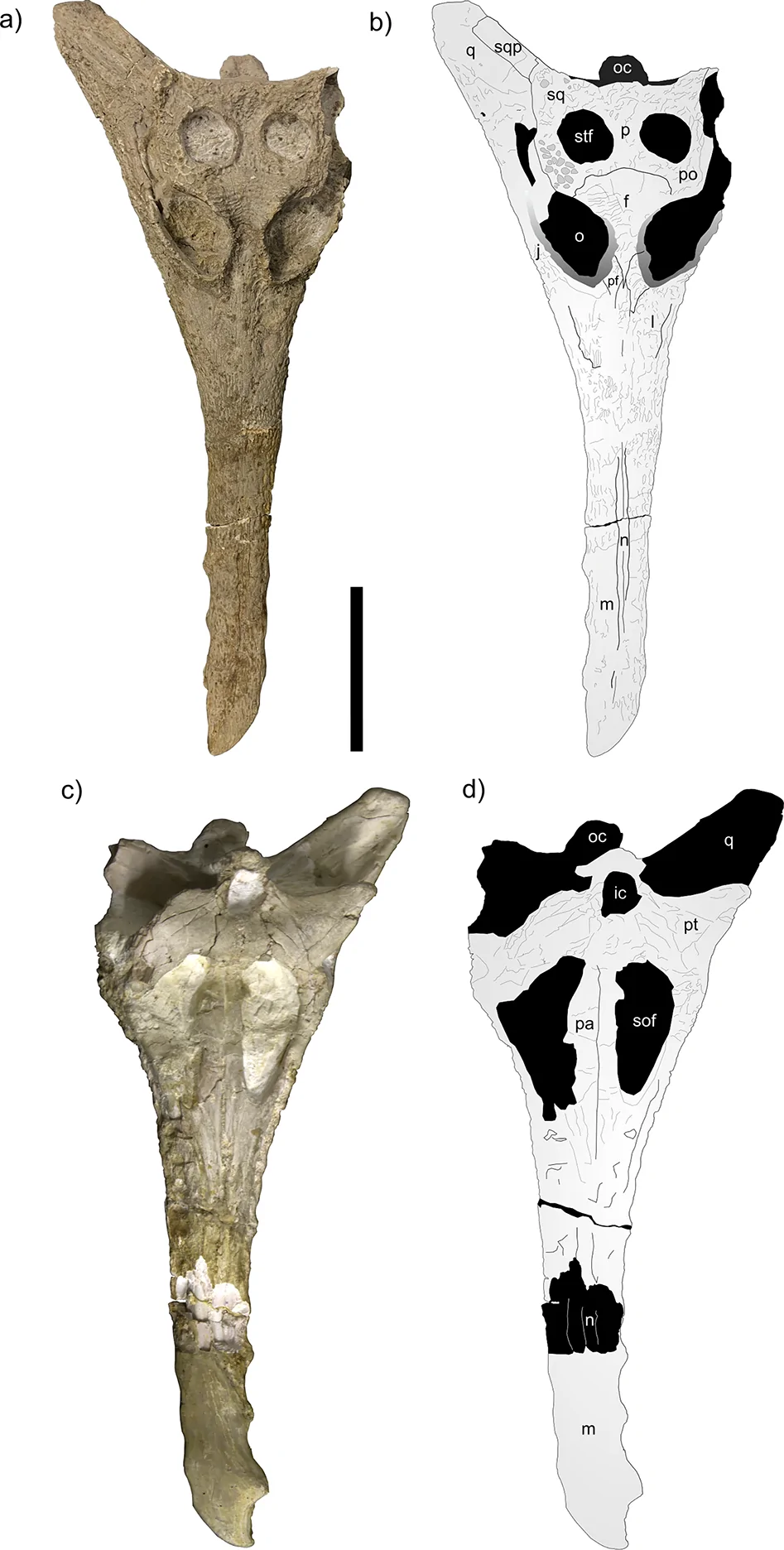
The holotype skull (SMNS 10575) of *H. cairensis* n. gen. n. comb. (a) Photograph of SMNS 10575 in dorsal view, (b) interpretative drawing of SMNS 10575 in dorsal view, (c) photograph of SMNS 10575 in ventral view and (d) interpretative drawing of SMNS 10575 in ventral view. Abbreviations: f, frontal; ic, internal choana; j, jugal; m, maxilla; ma, maxillary alveoli; n, nasals; o, orbit; oc, occipital condyle; p, parietal; pa, palatines; pf, prefrontal; po, postorbital; pt, pterygoid; q, quadrate; sof, suborbital fenestra; sq, squamosal; sqp, squamosal prong; stf, supratemporal fenestra. Scale bar, 10 mm.

**Figure 2.**
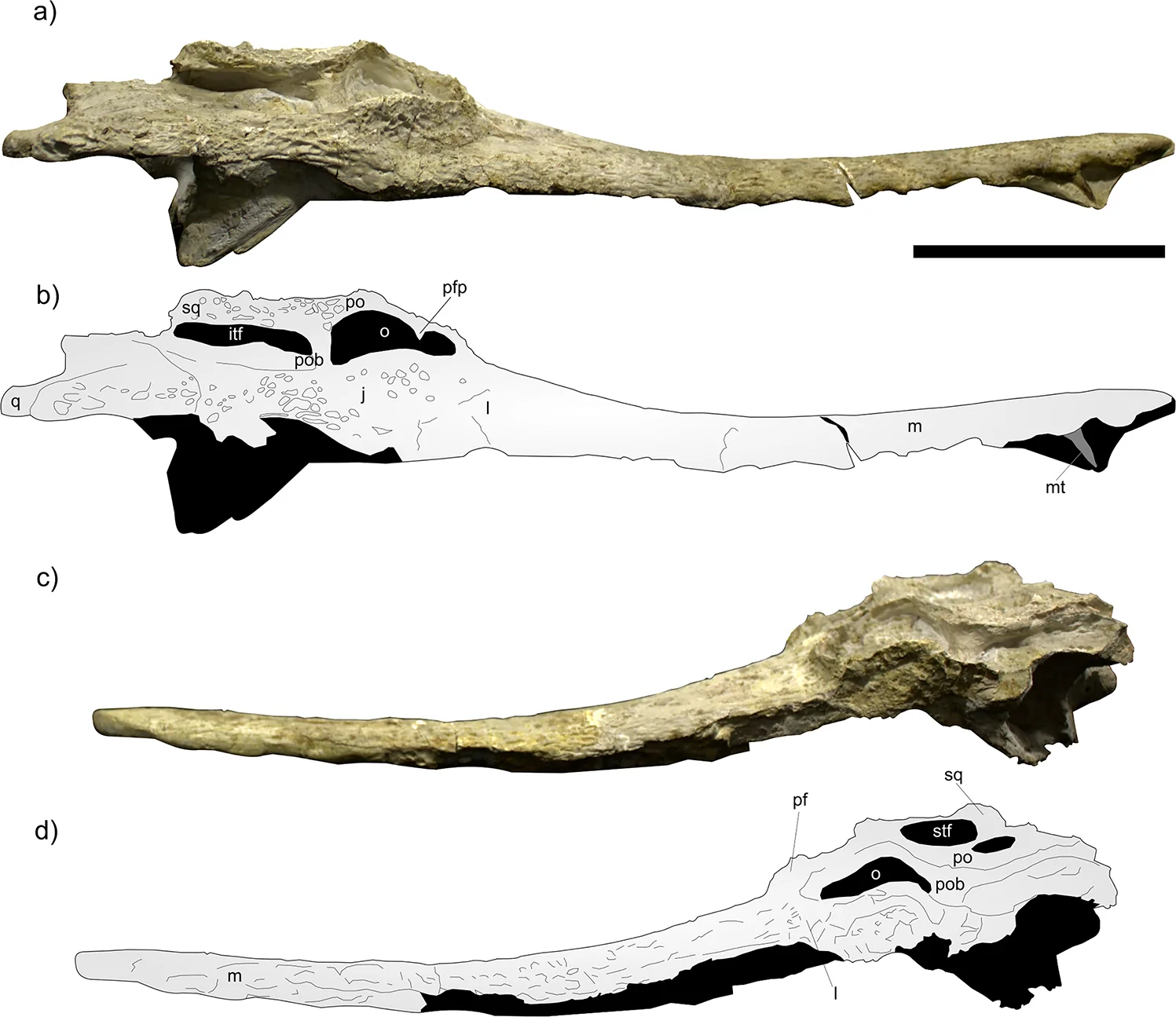
The holotype skull (SMNS 10575) of *H. cairensis* n. gen. n. comb. (a) Photograph of SMNS 10575 in right lateral view, (b) interpretative drawing of SMNS 10575 in right lateral view, (c) photograph of SMNS 10575 in left lateral view and (d) interpretative drawing of SMNS 10575 in left lateral view. Abbreviations: itf, intertemporal fenestra; j, jugal; l, lacrimal; m, maxilla; mt, maxillary tooth; o, orbit; pf, prefrontal; pfp, prefrontal pillar; po, postorbital; pob, postorbital bar; q, quadrate; sq, squamosal. Scale bar, 100 mm.

**Figure 3.**
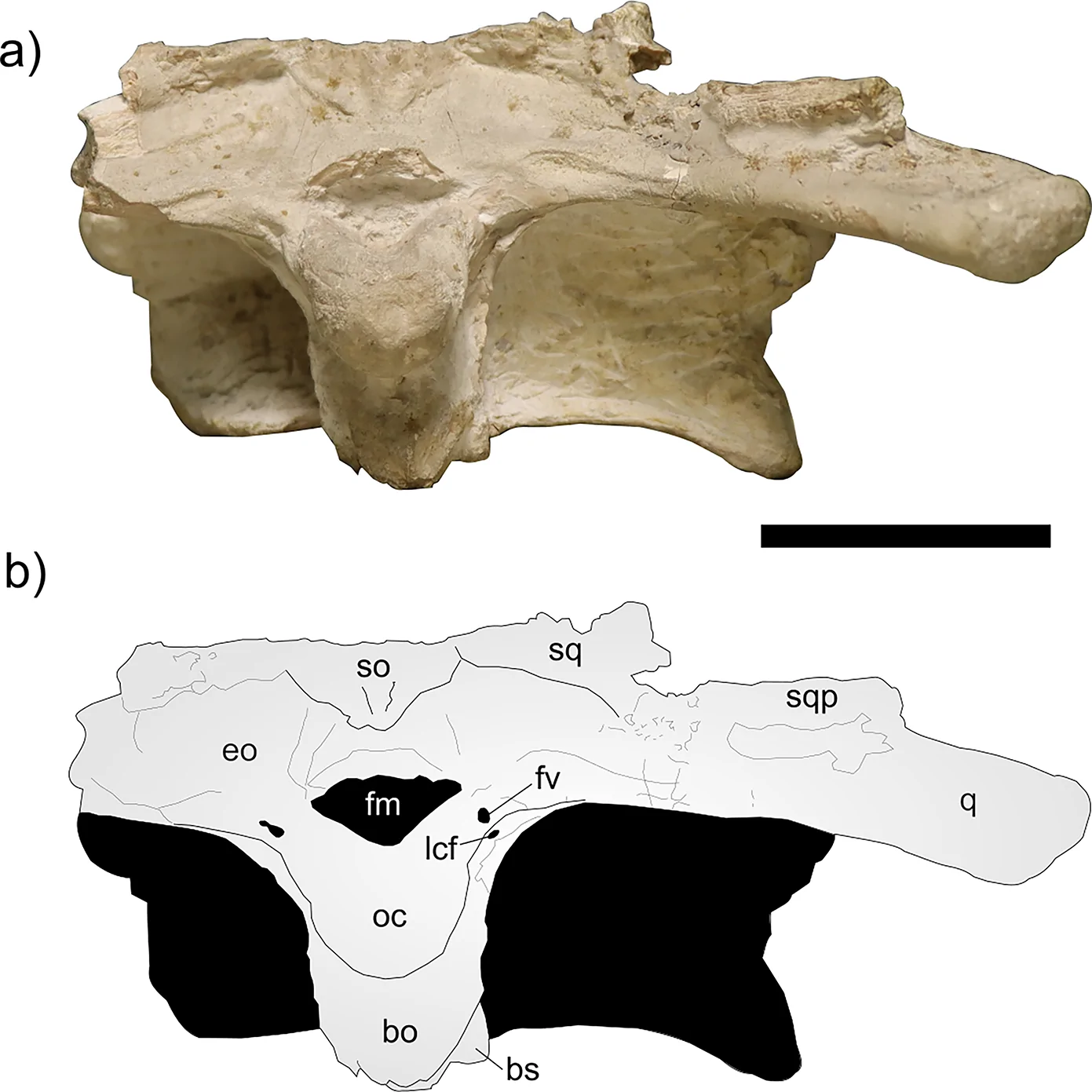
The holotype skull (SMNS 10575) of *H. cairensis* n. gen. n. comb. (a) Photograph of SMNS 10575 in posterior view and (b) interpretative drawing of SMNS 10575 in posterior view. Abbreviations: bo, basioccipital; bs, basisphenoid; eo, exoccipital; fm, foramen magnum; fv, vagus foramen; lcf, lateral carotid foramen; oc, occipital condyle; q, quadrate; so, supraoccipital; sq, squamosal; sqp, squamosal prong. Scale bar, 50 mm.

**Figure 4.**
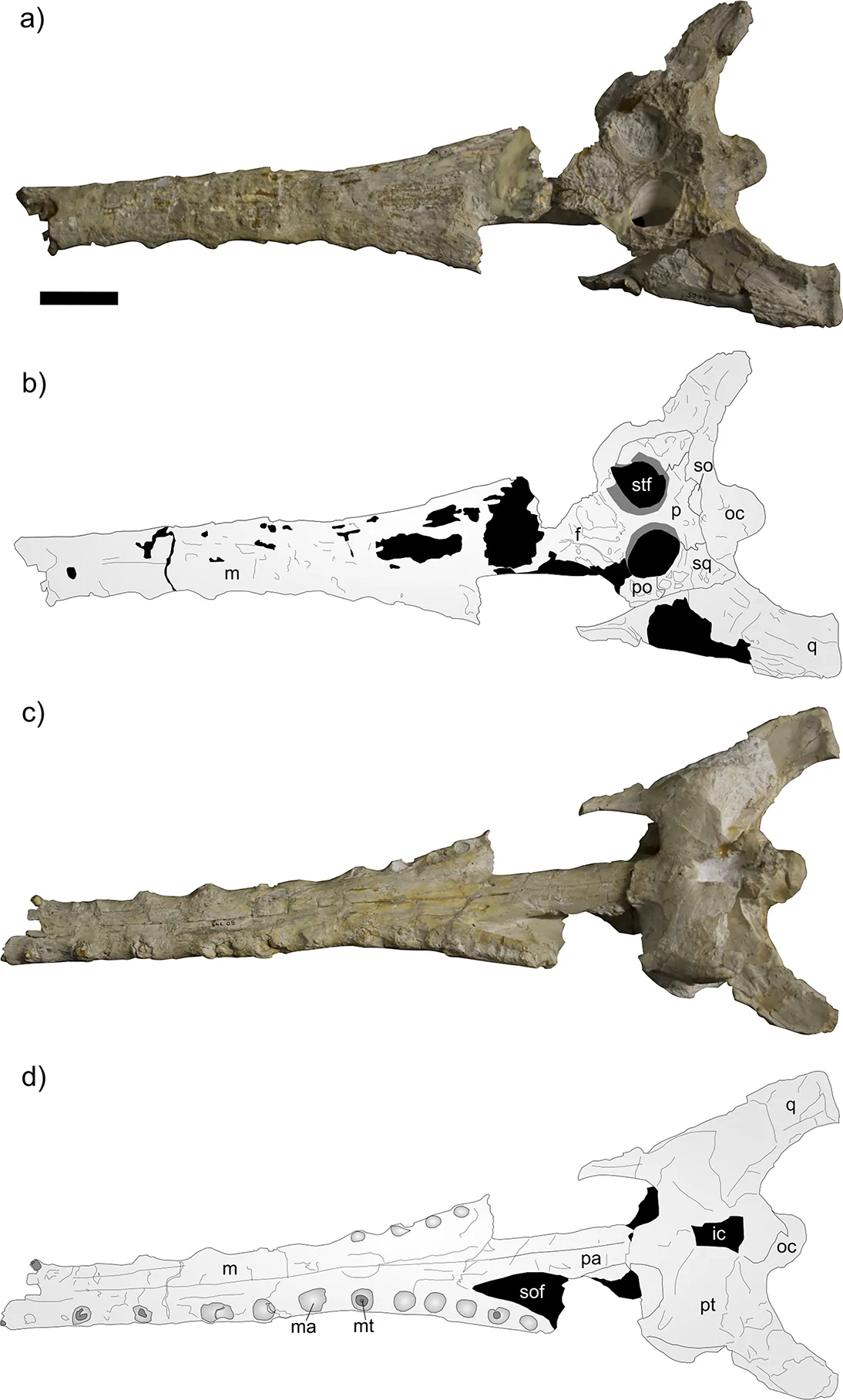
Referred skull (SMNS 50735) of *H. cairensis* n. gen. n. comb. (a) Photograph of SMNS 50735 in dorsal view, (b) interpretative drawing of SMNS 50735 in dorsal view, (c) photograph of SMNS 50735 in ventral view and (d) interpretative drawing of SMNS 50735 in ventral view. Abbreviations: f, frontal; ic, internal choana; m, maxilla; ma, maxillary alveoli; mt, maxillary tooth; oc, occipital condyle; p, parietal; pa, palatine; po, postorbital; pt, pterygoid; q, quadrate; so, supraoccipital; sof, suborbital fenestra; sq, squamosal; stf, supratemporal fenestra. Scale bar, 20 mm.

**Figure 5.**
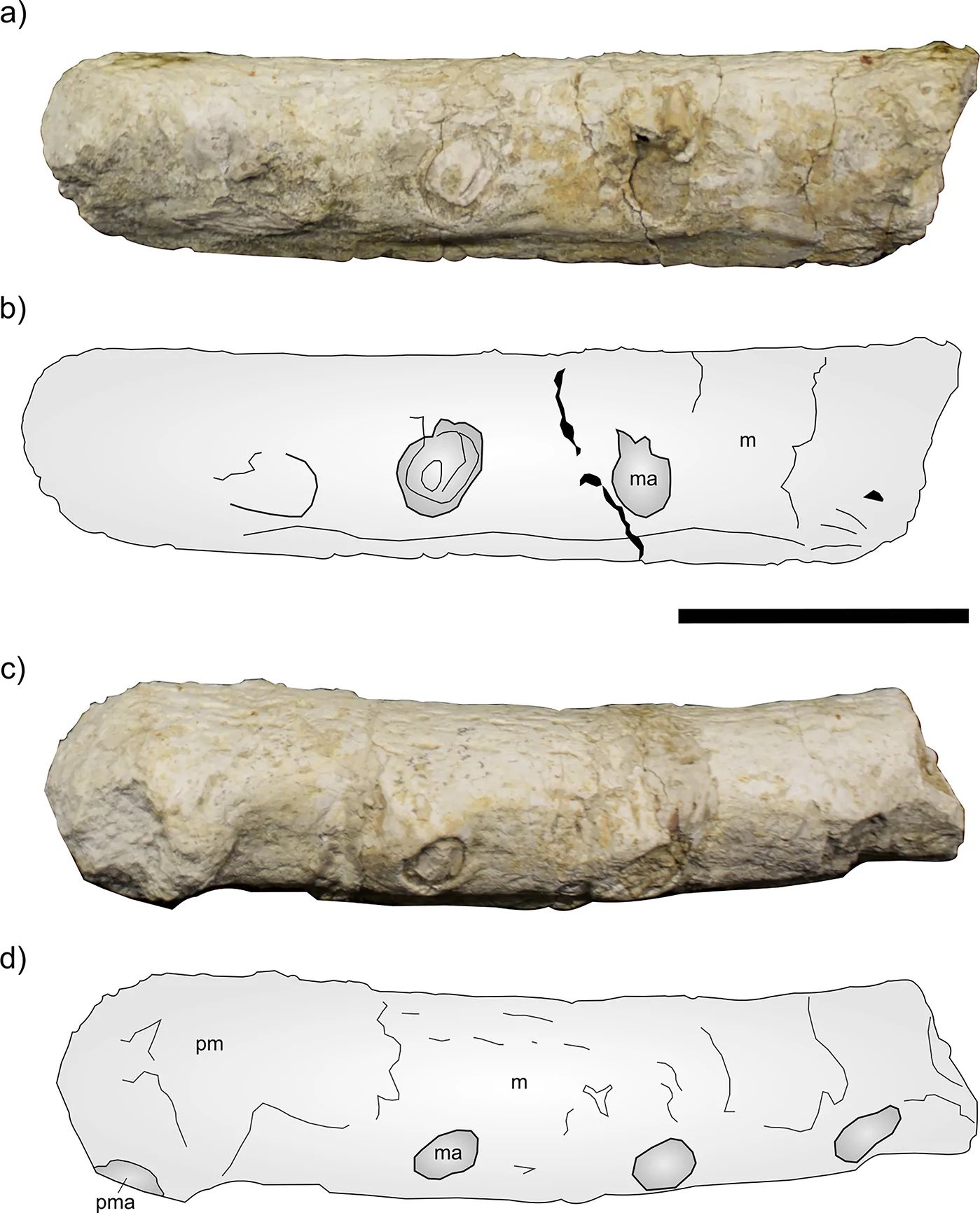
Referred premaxilla and maxilla (SMNS 50742) of *H. cairensis* n. gen. n. comb. (a) Photograph of SMNS 50742 in left lateral view, (b) interpretative drawing of SMNS 50742 in left lateral view, (c) photograph of SMNS 50742 in right lateral view and (d) interpretative drawing of SMNS 50742 in right lateral view. Abbreviations: m, maxilla; ma, maxillary alveoli; pm, premaxilla; pma, premaxillary alveoli. Scale bar, 50 mm.

**Figure 6.**
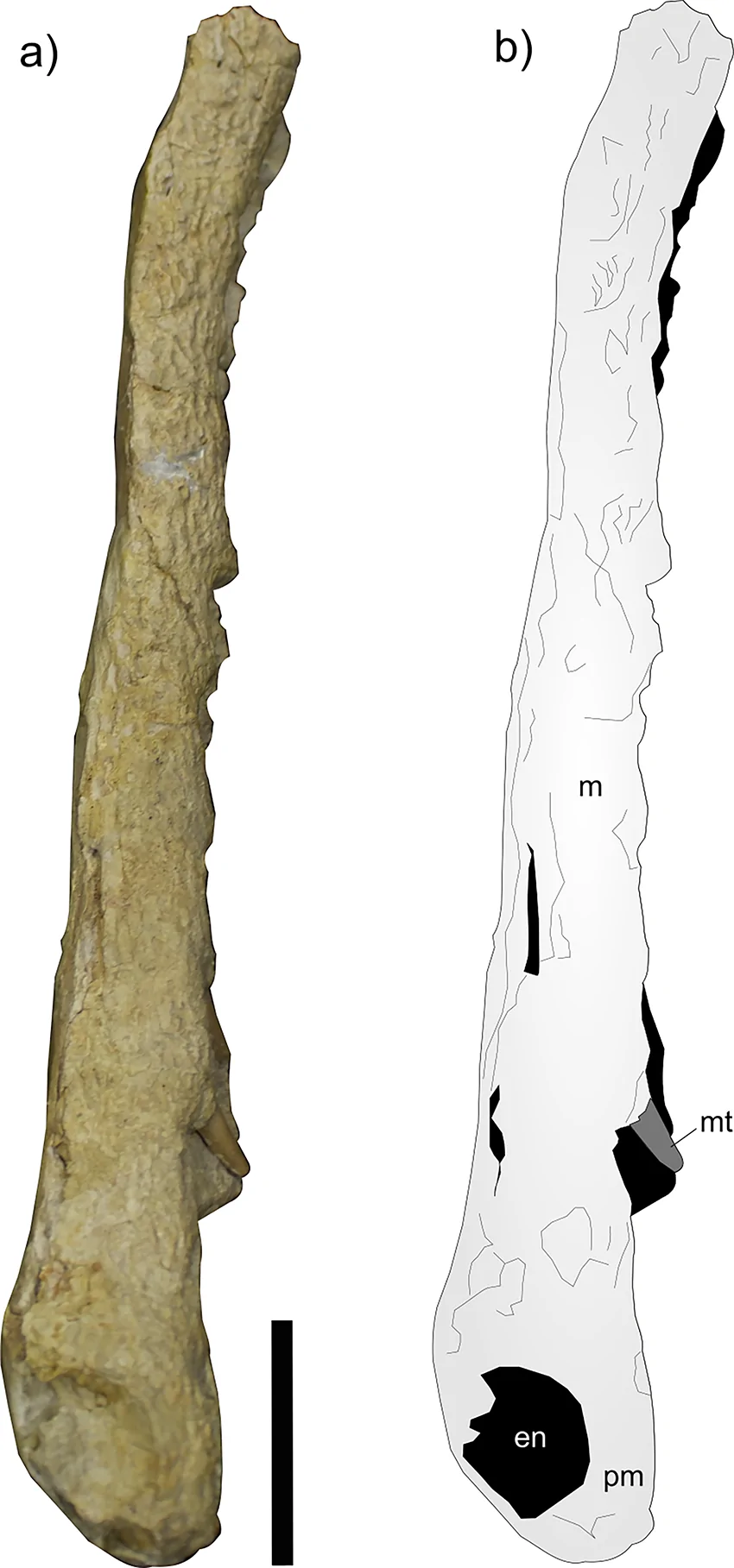
Referred left premaxilla and maxilla (SMNS 50743) of *H. cairensis* n. gen. n. comb. (a) Photograph of SMNS 50743 in dorsolateral view and (b) interpretative drawing of SMNS 50743 in dorsolateral view. Abbreviations: en, external naris; m, maxilla; mt, maxillary tooth; pm, premaxilla. Scale bar, 50 mm.

**Figure 7.**
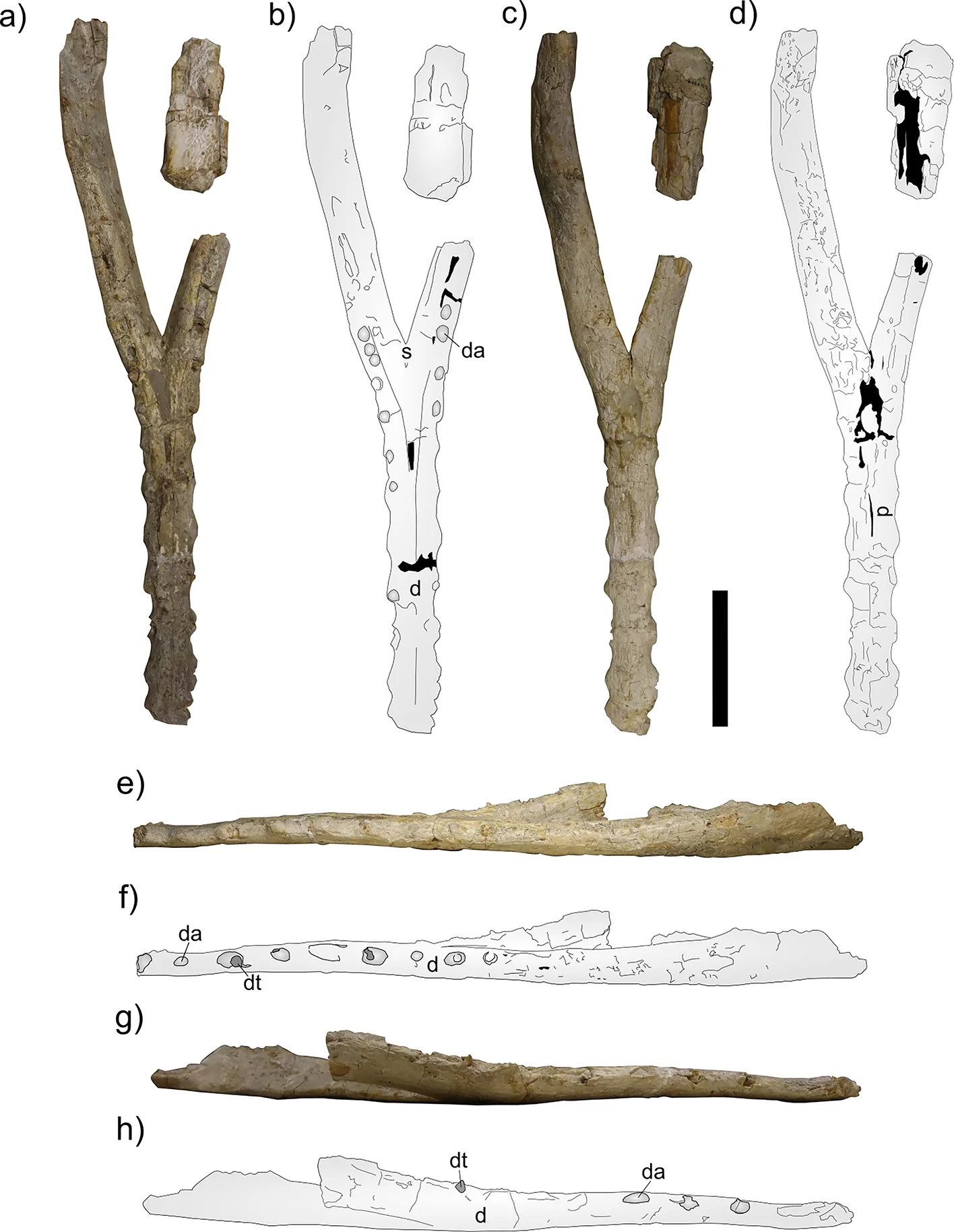
Referred mandible (SMNS 50739) of *H. cairensis* n. gen. n. comb. (a) Photograph of SMNS 50739 in dorsal view, (b) interpretative drawing of SMNS 50739 in dorsal view, (c) photograph of SMNS 50739 in ventral view, (d) interpretative drawing of SMNS 50739 in ventral view, (e) photograph of SMNS 50739 right lateral view, (f) interpretative drawing of SMNS 50739 in right lateral view, (g) photograph of SMNS 50739 left lateral view and (h) interpretative drawing of SMNS 50739 in left lateral view. Abbreviations: d, dentary; da, dentary alveoli; dt, dentary tooth; s, splenial. Scale bar, 100 mm.

**Figure 8.**
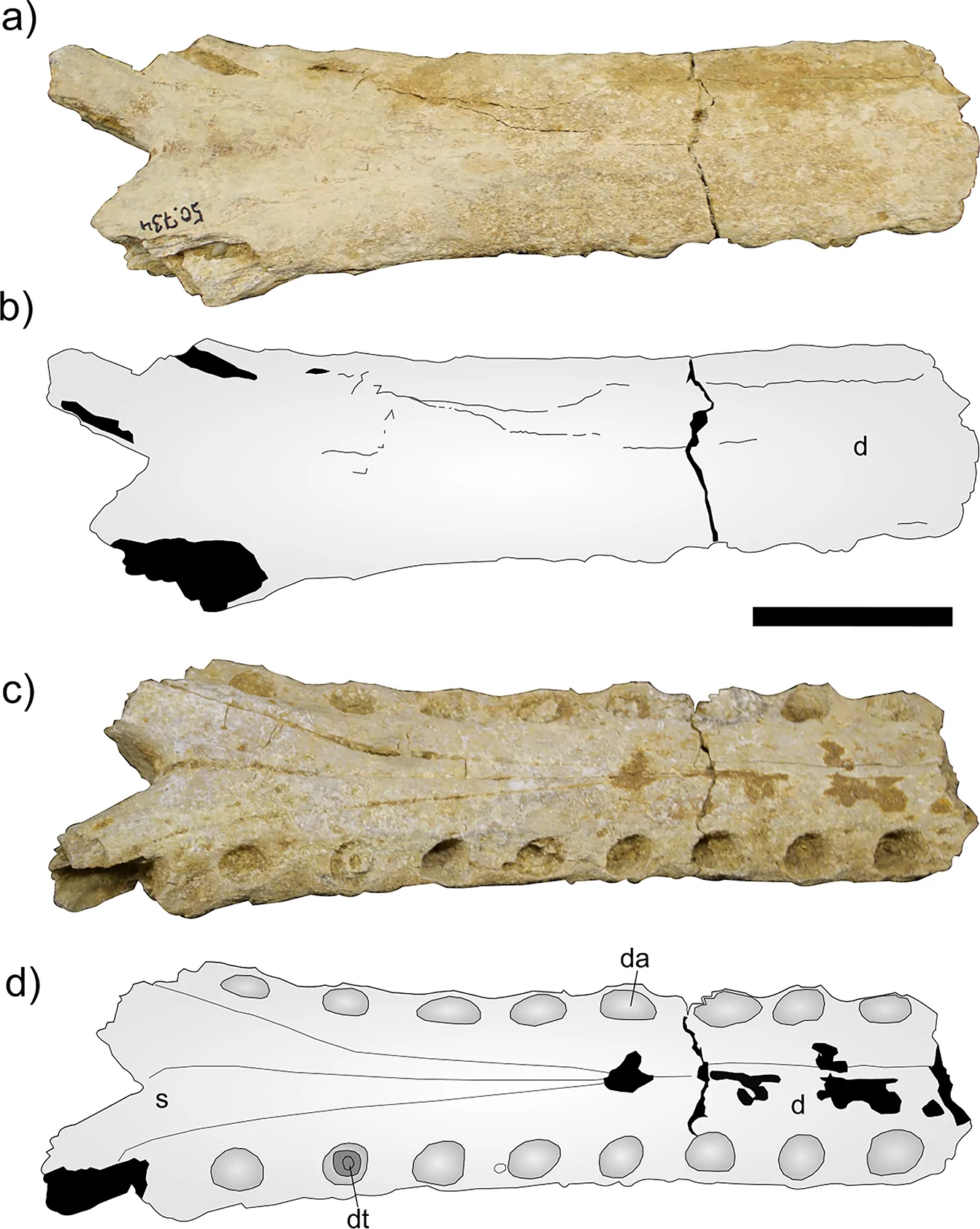
Referred partial mandible (SMNS 50734) of *H. cairensis* n. gen. n. comb. (a) Photograph of SMNS 50734 in ventral view, (b) interpretative drawing of SMNS 50734 in ventral view, (c) photograph of SMNS 50734 in dorsal view and (d) interpretative drawing of SMNS 50734 in dorsal view. Abbreviations: d, dentary; da, dentary alveoli; dt, dentary tooth; s, splenial. Scale bar, 50 mm.

### 3.1. Etymology

*Heru*, after the Egyptian god (also known as Horus), is often associated with, and possibly the inspiration for, the Great Sphinx of Giza [33]. The latter was sculpted from the Mokattam Formation [34], from which the type specimen of *H. cairensis* was recovered, with both sharing the absence of the anterior tip of their nose/snout. The genus suffix, -*gavialis*, reflects the relationship of *H. cairensis* to *Gavialis gangeticus* within Gavialoidea. Accordingly, the original species epithet, *cairense*, is modified to *cairensis* to agree with the masculine gender of *Herugavialis*.

### 3.2. Holotype

Müller [21] assigned SMNS 10575, a skull lacking the mandible, as the holotype of *T. cairense* (figures 1–3). This specimen is somewhat crushed, with the anterior part of the premaxillae missing, and the left side of the skull, particularly the orbit and postorbital region, damaged and incomplete [21].

### 3.3. Referred specimens

Müller [21] referred several specimens to *T. cairense*, all of which are of similar size and collected from the same area (and possibly same locality) as the holotype. As such, it is possible that some of these elements are from the same individual, including that of the holotype. Referred remains consist of (i) most of a second skull (SMNS 50735; figure 4); (ii) a partial snout comprising the posterior part of the premaxillae and anterior portion of the maxillae (SMNS 50742; figure 5); (iii) a fragment of the left premaxilla and maxilla (SMNS 50743; figure 6), which was stated by Müller [21] to be illustrated in fig. 3 of that publication; however, that figure depicts the mandible of *Tomistoma* (now *Eogavialis*) *gavialoides*; (iv) a partial mandible, described (but not figured) by Müller [21] as missing the anterior tip, as well as almost the entire right side and a large part of the left ramus, which is presumably SMNS 50739 given that it matches this description (figure 7); and (v) another partial mandible, preserving a portion of the dentaries (SMNS 50734, figure 8). Müller [21] also referred an occipital cranial segment to *T. cairense*, but this was not figured and could not be located in the SMNS collection. We follow Müller [21] in assigning SMNS 50734, 50735, 50739, 50742 and 50743 to *H. cairensis*.

### 3.4. Locality and horizon

The holotype and referred specimens were collected from Gebel Mokattam, east of Cairo, in northeastern Egypt [21]. They were recovered from the Mokattam Formation [21], which is a nummulitic marine limestone dated to the Lutetian, Middle Eocene [35].

### 3.5. Diagnosis

Within Gavialoidea, *H. cairensis* is characterized by a unique combination of characters: (i) lacrimal with an anteroposterior length more than twice that of the prefrontal; (ii) telescoped orbit formed by raised margins of the lacrimal, prefrontal and frontal; (iii) a squamosal wherein the dorsal and ventral margins do not diverge anteriorly; (iv) the distance between the distal tip of the paraoccipital process and the distal end of the quadrate condyle is greater than the maximum mediolateral width of the latter; and (v) a blunt dural peak on the encephalic endocast.

## 4. Description and comparisons of *Herugavialis cairensis*

### 4.1. Preservation

The holotype is the most completely preserved specimen of *Herugavialis*; however, it is missing the premaxillae, as well as the left jugal, quadratojugal and quadrate. Damage to the skull surface of the holotype means sutures are difficult to ascertain (such as that of the right jugal and quadratojugal), and ornamentation is mostly only seen on the right side of the skull, particularly on the skull table. The holotype is also characterized by a breakage in both maxillae (figure 1). The referred specimens are less completely preserved than the holotype, with SMNS 50735 missing the premaxillae, prefrontals and jugals, and partially preserving the maxillae, frontals and postorbitals (figure 4), SMNS 50734 preserving a partial dentary (figure 8), SMNS 50739 preserving a partial mandible (figure 7), and both SMNS 50742 and SMNS 50743 preserving a partial premaxilla and maxilla (figures 5 and 6). The sutures in the referred specimens are also difficult to ascertain owing to poor preservation of their external surfaces.

### 4.2. General shape

*Herugavialis* possesses a longirostrine skull, with an anteroposteriorly long and transversely narrow rostrum (figures 1 and 2). The snout forms approximately 64% of the total skull length, based on an estimation utilizing the combined anatomical information of SMNS 10575 and SMNS 50742. The mediolateral width remains consistent throughout the snout, gradually increasing posteriorly from the ninth maxillary alveolus to the skull table and quadrates. Along its lateral margins, the snout shows alveolar undulation (figures 1, 4 and 7).

The skull table of *Herugavialis* is planar across its full extent. This morphology characterizes several other gavialoids, namely *Aktiogavialis*, *Dollosuchoides*, *Eogavialis andrewsi*, *Eosuchus minor*, *Gavialosuchus*, *Gunggamarandu*, *Maomingosuchus*, *Maroccosuchus*, *Portugalosuchus*, *Thecachampsa*, ‘*Tomistoma*’ *gaudense*, ‘*Tomistoma*’ *lusitanica* and *Tomistoma schlegelii*, differing from the posterolateral edges of the skull table directed ventrolaterally from the sagittal axis in others [6,7,10,19,24,36–43].

### 4.3. Cranial openings

The external naris of *Herugavialis* can only be observed in one specimen, SMNS 50743, wherein it is incomplete (figure 6). Nevertheless, the shape of the external naris is clearly circular, contrasting with the heart-shaped naris seen in the gavialoids *Sutekhsuchus*, *Dollosuchoides* and *Eogavialis* [4,17,41]. There is no thin crest or fossa surrounding the external naris in *Herugavialis*, nor is there a notch posterolateral to the naris, both of which are also absent in all other gavialoids [10].

The orbits are large and elliptical, and, as in most gavialoids [10], the long axis of each orbit is oriented anteromedially to posterolaterally. Each orbit is bordered by the jugal laterally, the prefrontal and frontal medially, the lacrimal anteriorly and the postorbital posteriorly (figure 1). The margins of the orbits are telescoped, as in *Dadagavialis*, *Gavialis* and *Gryposuchus* [10,40,44–46].

The rounded supratemporal fenestrae are anteroposteriorly shorter than mediolaterally wide, with a ratio of 0.75. Each fenestra is bordered by the parietal medially, the postorbital anterolaterally and the squamosal posterolaterally. The rim of each fenestra is raised (figures 1 and 4), and the dermal bones of the skull roof do not overhang the rim of the fenestrae, consistent with the morphology seen in other gavialoids [10].

Only the left suborbital fenestrae are well preserved in SMNS 10575 (figure 1), with this specimen's right opening and both fenestrae in SMNS 50735 only partially preserved (figures 1 and 4). The suborbital fenestrae are anteroposteriorly longer than mediolaterally wide, with a sub-elliptical shape (figure 1). They are bordered by the maxillae anteriorly, the palatines medially and the pterygoids posteriorly.

### 4.4. Premaxilla

The premaxilla is partially preserved in both SMNS 50742 and SMNS 50743 (figures 5 and 6). As the premaxilla–maxilla suture is not visible, the extent of the dorsal process of the premaxilla relative to the maxillary alveolus cannot be determined. In addition, it is difficult to ascertain if any premaxillary alveoli are preserved in SMNS 50743 as the ventral surface is covered in matrix. In SMNS 50742, the premaxilla–maxilla suture is partially visible on the left side, with one premaxillary alveolus preserved (figure 5).

### 4.5. Maxilla

The maxilla is a dorsoventrally flat bone that comprises the majority of the rostrum. Both the dorsoventral height and the mediolateral width remain relatively consistent throughout the entirety of the maxilla. In SMNS 10575, the anteriormost maxillary alveoli are not preserved; however, several maxillary alveoli are preserved posteriorly, anterior to the palatines (figure 1), including one preserved tooth (figure 2). *Herugavialis* possesses at least 13 maxillary alveoli, since 13 and 7 alveoli are preserved on the right and left maxillae, respectively, in SMNS 50735 (figure 4). We estimate that *Herugavialis* had at least 15 alveoli on each maxilla. The alveoli are homodont, as in several other gavialoids, namely *Aktiogavialis*, *Dadagavialis*, *Eogavialis*, *Eosuchus*, *Eothoracosaurus*, *Gavialis*, *Gryposuchus*, *Ikanogavialis*, *Piscogavialis*, *Siquisiquesuchus* and *Thoracosaurus* ([10,25,37,40,46–51]). The alveoli are situated ventral to the maxillary palate, differing from the gavialoids *Dadagavialis*, *Eogavialis*, *Gavialis*, *Gryposuchus*, *Piscogavialis* and *Siquisiquesuchus*, where the alveoli are positioned dorsal to the maxillary palate [10,46,47,49,52].

### 4.6. Nasal

The nasals are preserved in both SMNS 10575 and SMNS 50739; however, the sutures between the nasals and the adjoining bones are best visible in SMNS 10575 (figure 1). The nasals are bordered by the maxillae laterally and the prefrontals and fused frontal posteriorly. In dorsal view, the nasals reduce in mediolateral width anteriorly; however, CT scan data reveal that they maintain their mediolateral width anteriorly, where they are concealed by the overlapping maxillae (figure 9). This differs from the nasals of extant gavialids, which reduce in mediolateral width anteriorly beneath the maxillae (figure 9). At the most posterior point of the nasals of *Herugavialis*, they are separated by a V-shaped projection of the frontal (figure 1).

**Figure 9.**
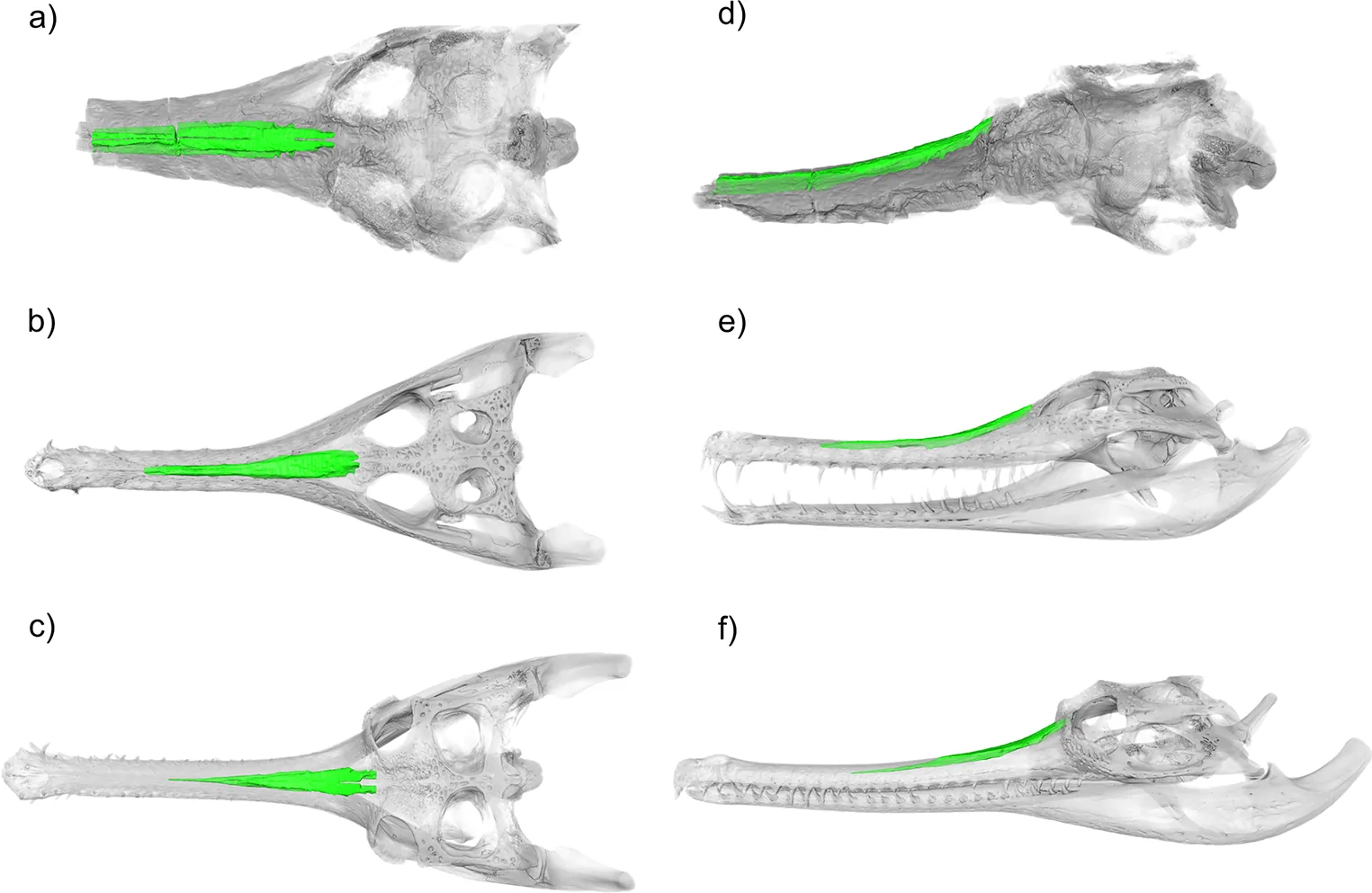
Comparison of the nasal bones with gavialids. CT scan reconstructions in dorsal view: *Herugavialis* (a), *Tomistoma* (b) and *Gavialis* (c); and lateral view: *Herugavialis* (d), *Tomistoma* (e) and *Gavialis* (f). Skulls not to scale.

### 4.7. Lacrimal

Owing to the preservation in this region, the shape and extent of the lacrimal cannot be fully determined from the dorsal surfaces of SMNS 10575 or SMNS 50739. However, from CT scans, the lacrimals in SMNS 10575 could be reconstructed (figure 10). The lacrimals form the anteromedial margins of the orbit and are separated from one another by the nasals, prefrontals and frontals. The anteroposterior length of the lacrimal is more than double that of the prefrontal. With the exception of *Kentisuchus*, the anteroposterior length of the lacrimal greatly exceeds that of the prefrontals in all gavialoids [4,10,53], although only *G. gangeticus* approaches the high ratio that characterizes *Herugavialis* (figure 10). As in *Gavialis*, the posterior margin of the lacrimal of *Herugavialis* is raised (figure 10).

**Figure 10.**
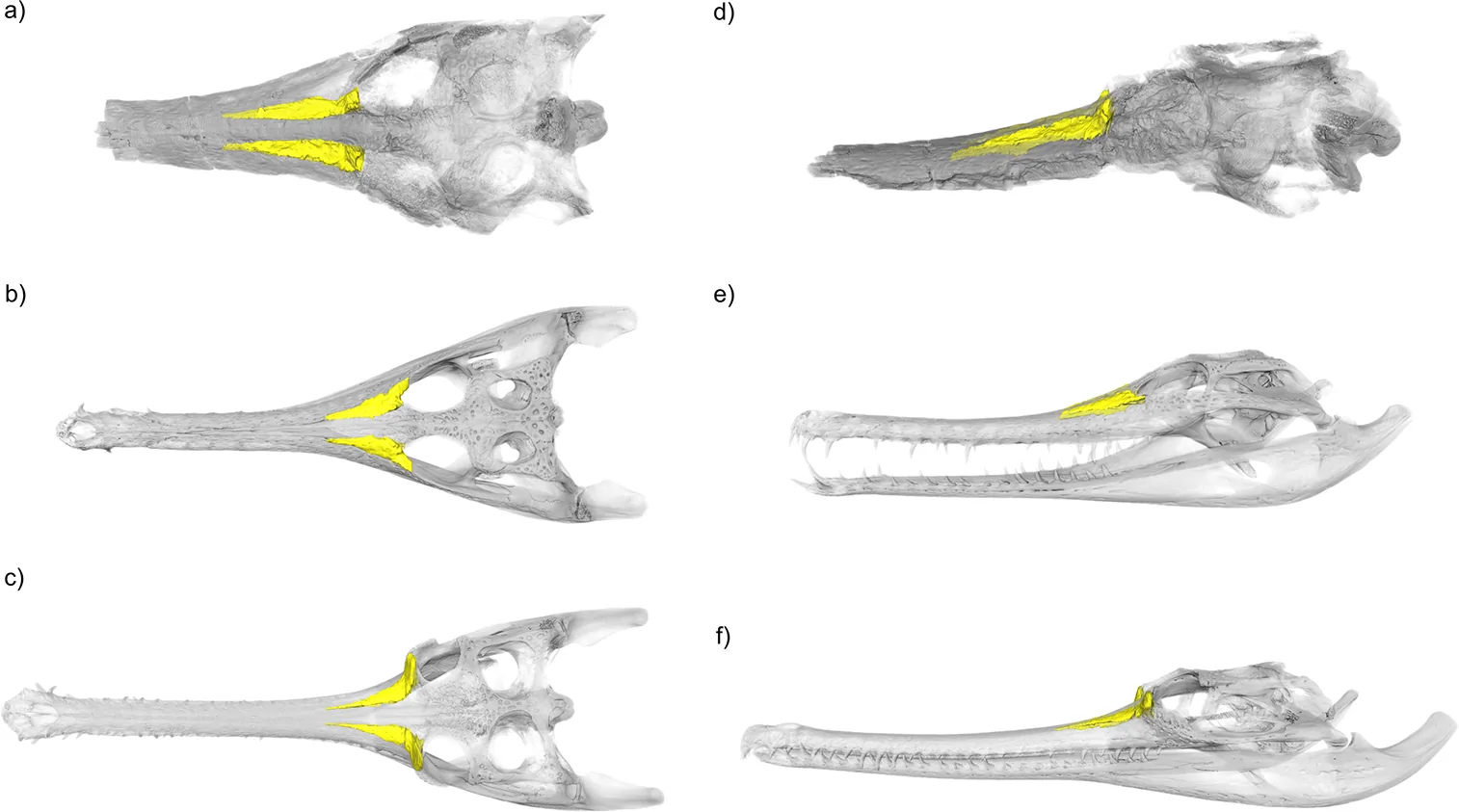
Comparison of the lacrimal bones with gavialids. CT scan reconstructions in dorsal view: *Herugavialis* (a), *Tomistoma* (b) and *Gavialis* (c); and lateral view: *Herugavialis* (d), *Tomistoma* (e) and *Gavialis* (f). Skulls not to scale.

### 4.8. Jugal

The extent of the jugal on the dorsal surface cannot be determined owing to poor preservation in both SMNS 10575 and SMNS 50739. Even with CT scan data, the sutural contact between the jugal and maxilla is difficult to determine in SMNS 10575. The ventral margin of the jugal arch is concave and lacks a ventrolateral sulcus. In addition, the lateral surface of the jugal is ornamented with sub-circular pits (figure 2). The anterior portion of the dorsal margin of the jugal, which forms the ventral margin of the orbit, slopes posteroventrally, gradually descending into the lower temporal bar (figure 2). This differs from both extant gavialids, in which the dorsal margin of the jugal is convex and continuous with that of the lower temporal bar in *T. schlegelii*, and steeply orientated in *G. gangeticus* [10].

### 4.9. Prefrontal

The prefrontals are anteroposteriorly elongate, with their greatest mediolateral width at their anteroposterior midpoint, being the narrowest in their anterior portion (figure 11). As in all other gavialoids [10], the prefrontals lack a thickened protuberance at the anteromedial orbital margin, are separated from one another by the frontal and nasals, and lack a linear sulcus lateral to the medial orbital margin. The anterior end of the prefrontal is positioned anterior to the anterior tip of the frontal, as in several other gavialoids, namely *Dollosuchoides*, *Eogavialis africanum*, *Eosuchus*, *Hanyusuchus*, *Kentisuchus*, *Maomingosuchus*, *Maroccosuchus*, *Penghusuchus*, *Thoracosaurus*, ‘*Tomistoma*’ *lusitanica*, *T. schlegelii* and *Toyotamaphimeia*, contrasting with all other gavialoids, in which the anterior end of the prefrontal is positioned posterior to, or at the same level as the anterior tip of the frontal [4,6,8,10,19,36,47,50,54]. CT scan data reveal that the prefrontal pillar internal morphology does not possess a pneumatic recess and is solid in *Herugavialis*, as in *E. africanum*, *Eosuchus*, *Gavialis*, *Paratomistoma*, *Portugalosuchus*, *Sutekhsuchus* and *T. schlegelii* [9,10,17,55]. The condition in all other gavialoids is uncertain, owing to lack of evaluation from CT scan data. As in *Gavialis*, the anterolateral margins of the prefrontals of SMNS 10575 are raised (figure 11).

**Figure 11.**
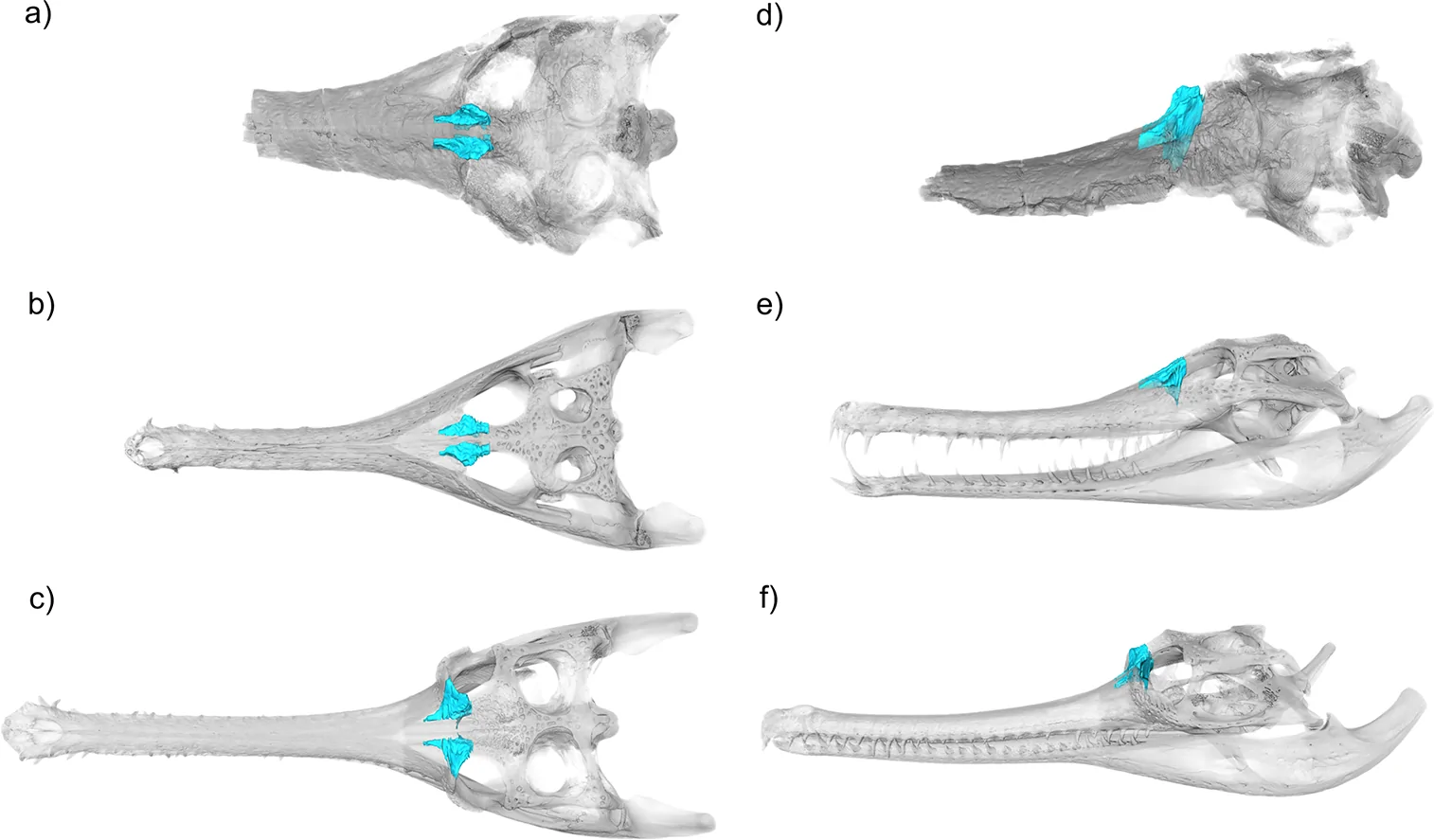
Comparison of the prefrontal bones with gavialids. CT scan reconstructions in dorsal view: *Herugavialis* (a), *Tomistoma* (b) and *Gavialis* (c); and lateral view: *Herugavialis* (d), *Tomistoma* (e) and *Gavialis* (f). Skulls not to scale.

### 4.10. Frontal

The paired frontals are fully fused and are anteroposteriorly longer than mediolaterally wide (figure 12). The frontals form the posteromedial margins of the orbits, and are bordered by the postorbitals laterally, the parietals posteriorly and the prefrontals and nasals anteriorly. The posteromedial margins of the frontals are upturned (figure 12), projecting into the orbit, similar to the morphology of *Gavialis* and *Gryposuchus* [44–46,56].

**Figure 12.**
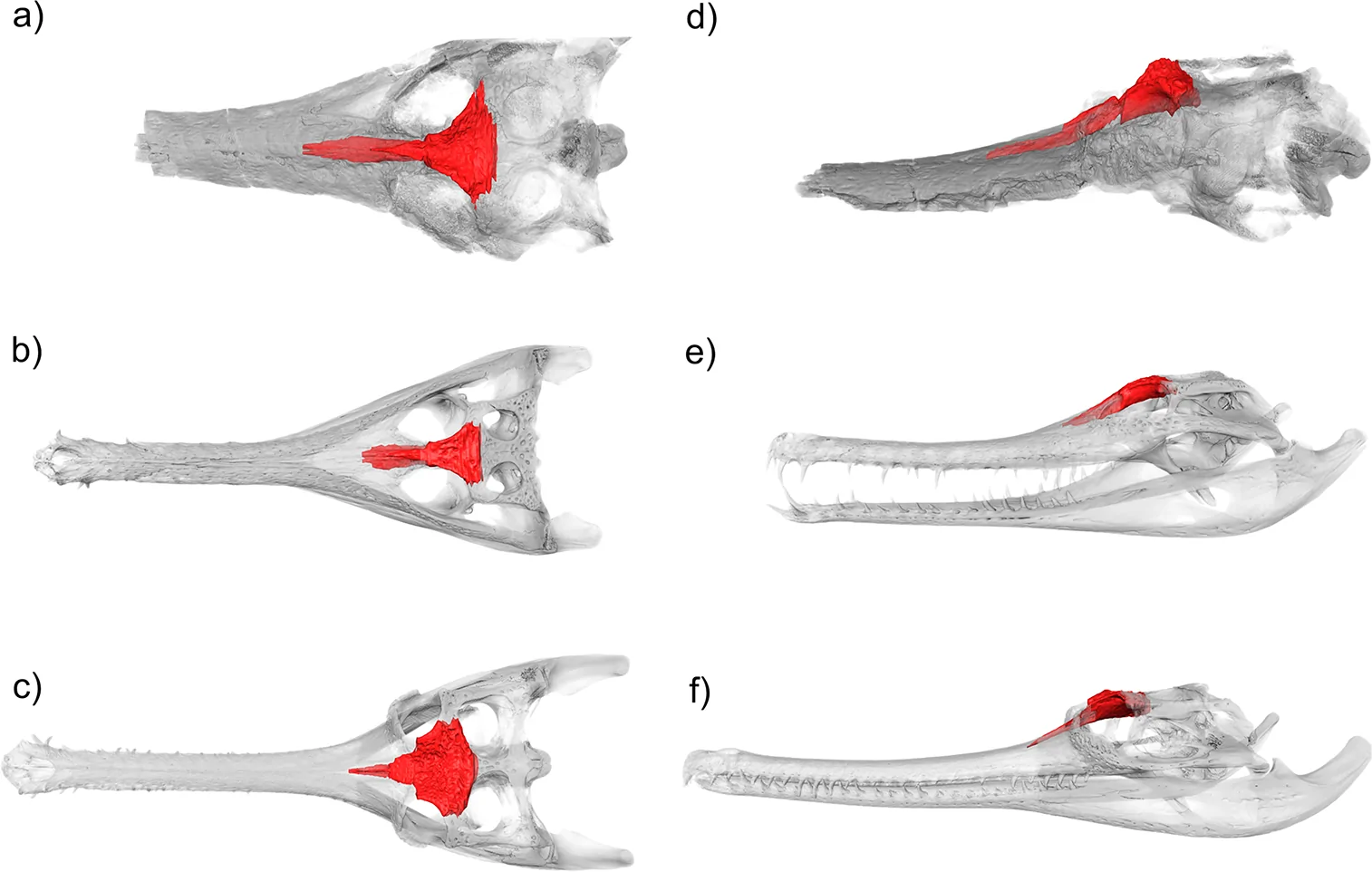
Comparison of the frontal bones with gavialids. CT scan reconstructions in dorsal view: *Herugavialis* (a), *Tomistoma* (b) and *Gavialis* (c); and lateral view: *Herugavialis* (d), *Tomistoma* (e) and *Gavialis* (f). Skulls not to scale.

Several ridges extend mediolaterally across the dorsal surface of the frontals of SMNS 10575 (figure 1), with SMNS 50735 also characterized by a series of pits (figure 4). As in many other gavialoids, except for *Aktiogavialis*, *Eosuchus minor*, *Eothoracosaurus*, *G. gangeticus*, *Gryposuchus*, *Ikanogavialis*, *Portugalosuchus* and *Thoracosaurus* ([10,25,37,38,40,45,46,48,56,57]; Salas-Gismondi *et al.* 51), the frontoparietal suture does not intersect the supratemporal fenestrae in *Herugavialis*. The frontoparietal suture is concavo-convex in *Herugavialis*, as in the gavialoids *Dollosuchoides*, *Eogavialis*, *Eosuchus*, *Gryposuchus pachakamue*, *Hanyusuchus*, *Paratomistoma*, *Piscogavialis*, *Sutekhsuchus*, *Thecachampsa* and *T. schlegelii* [4,10,17,24,37,41,43,47,50,52,54].

The anterior process of the frontals is posterior to that of the prefrontals. This corresponds with the morphology in other early diverging gavialoids, including several ‘thoracosaurs’, as well as *T. schlegelii*, whereas it extends anterior to the prefrontals in all other gavialids [9,10,25,50]. In addition, as in all other gavialoids, the anterior process of the frontals of *Herugavialis* is anterior to the anterior margin of the orbit. CT data reveal that the anterior tip of the frontals extends farther anteriorly under the ventral surface of the nasals (figure 12).

### 4.11. Postorbital

Both postorbitals are preserved in SMNS 10575 (figure 1), with only the left postorbital preserved in SMNS 50735 (figure 4). The postorbital is bordered by the frontal anteromedially, the parietal posteromedially, the squamosal posteriorly and the jugal ventrolaterally. The postorbital forms part of the posterior margin of the orbit, but, unlike the contribution from the prefrontal and frontal, the postorbital margin is not upturned (figures 1 and 4). Heavy pitting characterizes the right postorbital of SMNS 10575, whereas no pitting can be observed on the left postorbitals of SMNS 10575 and SMNS 50735, although this might be due to poor preservation (figures 1 and 4). The postorbital bar is laterally inclined, and thus visible in dorsal view. It is anteroposteriorly expanded and elliptical in cross-section (figure 1), as is the case in most other gavialoids, including *G. gangeticus*, but differing from *T. schlegelii*, *Gavialosuchus eggenburgensis* and *P. courti*, wherein the postorbital bar is mediolaterally reduced and is circular in cross-section [10,24,42].

### 4.12. Parietal

The fused parietals are preserved in both SMNS 10575 (figure 1) and SMNS 50735 (figure 4). They are bordered by the frontals anteriorly, the postorbitals laterally and the squamosals posterolaterally. The parietals form the raised medial margins of the supratemporal fenestrae (figures 1 and 4). As in all other gavialoids [10], there is no sagittal crest on the dorsal surface between the supratemporal fenestrae, nor are there any foramina on the medial walls of the supratemporal fenestrae in *Herugavialis*. The maximum mediolateral width of the parietals is positioned between the supratemporal fenestrae and the fronto-parietal suture, with the minimum mediolateral width occurring between the supratemporal fenestrae. The sutures between the parietals and squamosals are only partially visible in SMNS 10575 (figure 1), but, based on SMNS 50735, they are orientated anteroposteriorly in a ‘zig-zag’ shape (figure 4).

### 4.13. Squamosal

The dorsal and ventral margins of the squamosal are parallel (figure 1). This differs from the condition in most gavialoids, in which these margins diverge from one another anteriorly [10]. Among gavialoids, only *Eosuchus lerichei*, *Maomingosuchus* and *T. schlegelii* display the same morphology as *Herugavialis* [10,19,50,58]. The angle between the dorsal margins of the paroccipital process and cranial table is approximately 40° in *Herugavialis*, which is similar to the condition seen in most other gavialoids, although this angle is less than 10° in several South American species [10]. The posterolateral prong of the squamosal of *Herugavialis* is elongate, greater than half the anteroposterior length of the cranial table; in this regard, it is similar to ‘*Tomistoma*’ *lusitanica*, as well as several Central and South American gavialoids, including *Aktiogavialis*, *Piscogavialis* and *Siquisiquesuchus* [10,36,46,49,52].

### 4.14. Quadrate

In SMNS 10575, the right quadrate is well preserved, but the left quadrate is missing (figure 1). Both quadrates are preserved in SMNS 50735 (figure 4). The quadrate projects posteroventrally and is positioned ventral to the squamosal and lateral to the otoccipital. A small foramen aereum, which is less than half the dorsoventral height of the quadrate hemi-condyle, is situated on the dorsomedial corner of the quadrate ramus. The quadrate condyle is sub-rectangular, with no notch on the dorsal articular border, at least in SMNS 10575; it is not possible to determine if this notch is present in SMNS 50735 because of poor preservation in this region. The dorsal and ventral margins of the quadrate condyle in SMNS 10575 are sub-parallel, similar to the condition in *G. gangeticus*, several gryposuchines, *Hanyusuchus* and *Aktiogavialis* [10,46,54].

### 4.15. Supraoccipital

The sub-triangular supraoccipital is exposed on the dorsal surface of the skull table. The extent of exposure is difficult to determine in SMNS 10575 as the supraoccipital-parietal suture is not well preserved (figure 1). However, in SMNS 50735, the mediolateral width of the supraoccipital is less than half that along the posterior margin of the parietal (figure 4). Exposure of the supraoccipital on the dorsal surface is seen in most gavialoids, except for *Eosuchus minor*, *Gryposuchus croizati*, *Hanyusuchus*, *Thecachampsa*, ‘*Tomistoma*’ *gaudense* and ‘*Tomistoma*’ *lusitanica* [7,10,36,43,46,48,54]. The posterolateral tuberosities of the supraoccipital of *Herugavialis* are not visible in dorsal view, as is also the case in *Aktiogavialis*, *Eosuchus lerichei*, *Gavialosuchus*, *Maomingosuchus*, *Maroccosuchus*, *Sutekhsuchus* and *Thoracosaurus isorhynchus*, differing from the two extant gavialoids [6,10,17,19,25,40,42,50]. As in other gavialoids [59], *Herugavialis* possesses a nuchal crest on the supraoccipital (figure 3). Only the most ventral part of the nuchal crest is visible, with the dorsalmost part difficult to distinguish. However, this is probably to be a preservational artefact (figure 3). As a result, the postoccipital processes of the supraoccipital cannot be accurately identified in *Herugavialis*.

### 4.16. Exoccipital

The occiput of the exoccipital is inclined posteriorly, and therefore visible in dorsal view (figures 1 and 4). This reflects the morphology seen in several other gavialoids, namely *Gavialis*, *Gryposuchus*, *Ikanogavialis*, *Piscogavialis*, *Portugalosuchus*, *Siquisiquesuchus* and *Thoracosaurus neocesarensis*, whereas the occiput is not visible in dorsal view in all other members of the clade [10,38,46,48,49,52,57]. As in all other crocodylians [60], the carotid foramina are orientated at the same level as the occipital condyle in *Herugavialis* (figure 3). In addition, the lateral carotid foramen and the vagus foramen are positioned close to one another, differing from the morphology seen in several other gavialoids, namely *Sutekhsuchus*, *Thoracosaurus isorhynchus* and *Piscogavialis laberintoensis* [13,17,25], where there is a larger separation between them.

### 4.17. Basioccipital and basisphenoid

The basioccipital forms the ventromedial margin of the foramen magnum and the posterior wall of the median pharyngeal foramen (figure 3). It is ventral to the occipital condyle and is only visible in posterior view. The mediolateral width of the basioccipital tubera is less than twice that of the occipital condyle, contrasting with the condition seen in *Gavialis* and *Gryposuchus* ([10,46]; Salas-Gismondi *et al.* [51]). The dorsoventral height of the basioccipital of *Herugavialis* is greater than that of the occipital condyle, as in the gavialoids *Eosuchus minor*, *Eothoracosaurus*, *Gavialosuchus*, *Ikanogavialis*, *Kentisuchus*, *Maomingosuchus*, *Maroccosuchus*, *Penghusuchus*, *Thecachampsa sericodon*, *Thoracosaurus*, ‘*Tomistoma*’ *lusitanica* and *T. schlegelii* [4,6,10,19,36,42,43,48,50,57,61]. The contact between the exoccipital and basioccipital could not be determined. The lateral margins of the basioccipital are parallel to each other, which is the condition seen in most gavialoids, except for *Aktiogavialis*, *Eogavialis*, *Gavialis*, *Gryposuchus neogaeus* and *Piscogavialis* [10,40,44,47,51]. As in all other gavialoids, the basioccipital in *Herugavialis* is orientated vertically [10]. *Herugavialis* lacks a sagittal crest on the ventral surface of the basioccipital, while *Gavialis*, *Gryposuchus*, *Piscogavialis*, *Sutekhsuchus* and *Toyotamaphimeia* possess a sagittal crest ([8,10,17,44–46,52,56]; Salas-Gismondi *et al.* [51]). In addition, *Herugavialis* lacks a concavity on the ventral margin of the basioccipital, while *Aktiogavialis*, *Gavialis*, *Gryposuchus*, *Ikanogavialis*, *Piscogavialis* and *Portugalosuchus* possess this concavity ([10,38,40,44–46,52,56]; Salas-Gismondi *et al.* [51]). Ventrolateral to the basioccipital, the basisphenoid is only partially exposed in posterior view (figure 3).

### 4.18. Laterosphenoid and prootic

Both the laterosphenoid and the prootic are preserved in SMNS 10575 and SMNS 50735. However, as a result of matrix infilling of the supratemporal fenestrae, neither element is exposed (figures 1 and 4). The exception to this is the left supratemporal fenestra of SMNS 50735, but the suture between the laterosphenoid and prootic is not preserved here. The suture can also not be discerned from the CT scan data.

### 4.19. Palatine

The anterior process of the palatine extends beyond the anterior margin of the suborbital fenestra by the distance of three maxillary alveoli (figures 1 and 4). This reflects the morphology in *Gavialis*, as well as several other gavialoids, but differs from that of *T. schlegelii*, as well as *Dollosuchoides*, *Eosuchus*, *Gavialosuchus*, *Hanyusuchus*, *Kentisuchus*, *Maomingosuchus*, *Maroccosuchus* and *Penghusuchus*, wherein the anterior process extends to the distance of two alveoli or fewer [4,6,10,19,50,54,61]. The maxilla–palatine suture is situated level with the anterior margin of the suborbital fenestra, as in *Eosuchus*, *Gavialis*, *Gryposuchus*, *Ikanogavialis*, *Piscogavialis*, *Thecachampsa*, *Thoracosaurus*, *Toyotamaphimeia* and ‘*Tomistoma*’ *lusitanica* [8,10,25,36,43,46,48,50,52,57].

### 4.20. Pterygoid

The pterygoids are preserved in both SMNS 10575 and SMNS 50735 (figures 1 and 4). They entirely surround the internal choanae, which are elliptical. This morphology is similar to the condition in most other gavialoids, except for *Gavialis lewisi*, *Penghusuchus*, *Thecachampsa* and ‘*Tomistoma*’ *lusitanica*, wherein the choanae are sub-triangular [10,36,43,61,62]. The pterygoid of *Herugavialis* is unornamented and flush with the margins of the choanae, as in most other gavialoids, except *Gavialis lewisi*, *Maomingosuchus* and *Piscogavialis*, wherein the choanal margin is elevated relative to the pterygoid [10,19,52,62].

### 4.21. Mandible

The dentary is partially preserved in both SMNS 50734 and SMNS 50739 (figures 7 and 8), missing the anteriormost portion. At least 12 alveoli are present along the preserved portion. The dentary alveoli are homodont, and only one tooth is preserved (figure 7). In most gavialoids, the fourth dentary alveolus is the largest [10]. A second enlarged alveolus, posterior to the fourth alveolus, is also present in *Hanyusuchus*, *Maroccosuchus*, *Penghusuchus*, *Thecachampsa antiquus* and ‘*Tomistoma*’ *lusitanica* [6,36,43,54,61]. As *Herugavialis* shows homodonty in its dentary alveoli, we assume that the fourth dentary alveolus is not preserved, and therefore at least the first four dentary alveoli are missing (figure 7). As such, we infer that *Herugavialis* was characterized by at least 16 alveoli on each dentary. In lateral view, the dorsal profile of the dentary is linear (figure 7). This condition is seen in most gavialoids, particularly between the fourth and tenth dentary alveoli [10], except for *Eosuchus minor*, *Maroccosuchus* and *Penghusuchus*, wherein the dorsal profile is concave [6,48,61]. Of the alveoli preserved, the mandibular symphysis extends to approximately 10 dentary alveoli, as in *Dollosuchoides*, *Eosuchus minor*, *Maomingosuchus*, *Maroccosuchus*, *Sutekhsuchus*, *Thecachampsa*, ‘*Tomistoma*’ *calaritanum* and ‘*Tomistoma*’ *lusitanica* [4,6,7,17,19,36,48]. The posteriormost dentary alveoli are orientated in a straight line (figure 7), as in all other gavialoids [10].

As seen in all other gavialoids [10], the splenial fully participates in the symphysis and is dorsal and ventral to the Meckelian fossa in *Herugavialis*. The anteriormost part of the splenial extends to the distance of seven dentary alveoli in *Herugavialis*. This condition is also present in most gavialoids, except for *Dollosuchoides* and *Maroccosuchus*, wherein the splenial extends to the distance of two to three dentary alveoli [4,6], and *Eogavialis*, *Gavialis*, *Ikanogavialis* and *Siquisiquesuchus*, in which the splenial extends to the distance of more than seven dentary alveoli [10,47,49,57]. In most gavialoids, including *Herugavialis*, the splenial–dentary suture is a constricted, narrow V-shape adjacent to the dentary toothrow in dorsal view. By contrast, the splenial–dentary suture is a straight, and considerably wider, V-shape in *Eosuchus*, *Gavialis*, *Gryposuchus croizati*, *Piscogavialis* and *Sutekhsuchus* [10,17,46,50,52]. In *Herugavialis*, the splenial lacks the additional anterior process that is present in *Gavialis* [10].

### 4.22. Dentition

All alveoli in the upper and lower jaw are circular in cross-section. The few teeth that are preserved (figures 2, 6 and 7) are long and slender, with a rounded apex, similar to that of other gavialoids, such as *E. lerichei* [50], *P. laberintoensis* [13] and *T. isorhynchus* [25]. The medial and lateral surfaces of the teeth are smooth and can be differentiated by carinae.

### 4.23. Encephalic endocast

The encephalic endocast of *Herugavialis* (figure 13) shows greater curvature than that of most gavialoids [63]. This is exemplified by the cephalic (132°) and pontine (153°) flexure angles, which are measured by the same protocol as in Burke & Mannion [64], and are most similar to the values observed in *T. schlegelii* and crocodyloids [64]. The cerebrum of *Herugavialis* has its greatest transverse width at its most posterior point, reducing in width anteriorly (figure 13). The distinction between the olfactory tract and olfactory bulb can be identified by a slight increase in the transverse width of the latter relative to the former; this lack of clear osteological division has also been noted in *Gavialis* and *E. lerichei* [9]. In *Herugavialis*, the anteroposterior length of the olfactory bulb is less than half that of the olfactory tract, as in other gavialoids [9,63,65]. The encephalic endocast of *Herugavialis* is characterized by a blunt dural peak, which is not seen in any other gavialoid, but is present in some other crocodylians, including the mekosuchine *Trilophosuchus rackhami* [66]. The pituitary fossa in *Herugavialis* is characterized by two channels that would have housed the cerebral carotid arteries, which curve dorsolaterally at their most posterior point, as in other gavialoids [9]. The endosseous labyrinth of *Herugavialis* could not be reconstructed because of the low quality of the scan in this region of the skull.

**Figure 13.**
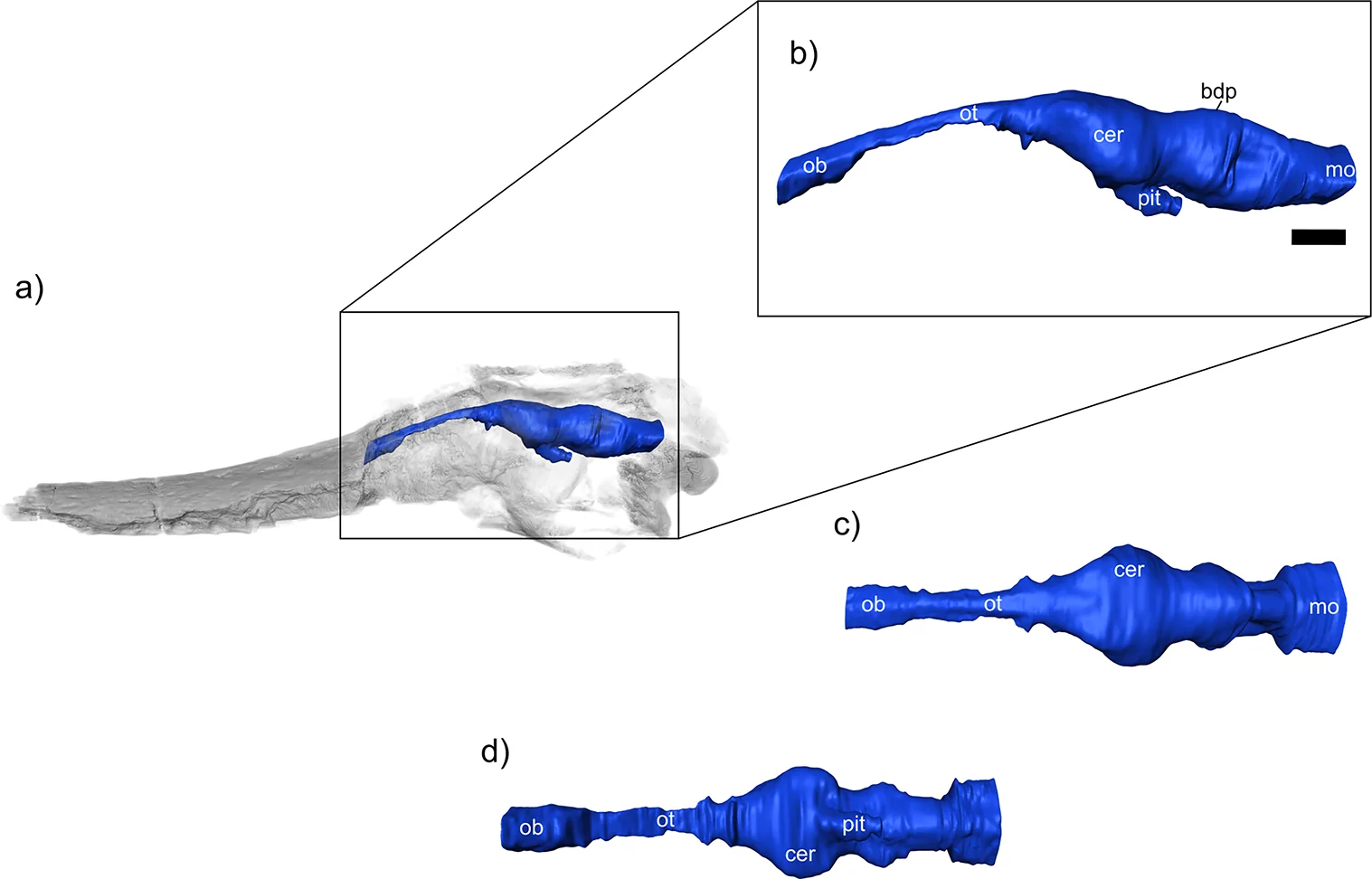
Encephalic endocast of the holotype skull (SMNS 10575) *H. cairensis* n. gen. n. comb. (a) Skull rendering in left lateral view with encephalic endocast in blue, (b) encephalic endocast in left lateral view, (c) dorsal view and (d) ventral view. Abbreviations: bdp, blunt dural peak; cer, cerebrum; mo, medulla oblongata; ob, olfactory bulb; ot, olfactory tract; pit, pituitary fossa. Scale bar, 10 mm.

### 4.24. Paratympanic sinus system

The median pharyngeal canal is situated on the basisphenoid–basioccipital suture and separates into two branches dorsally (figure 14). As in other gavialoids [9,64], the pharyngeal intersection forms a U-shape in lateral view. The basisphenoid branch of the median pharyngeal canal forms the basisphenoid recess, which is located ventral to the cerebral carotid arteries. The basioccipital branch of the median pharyngeal canal forms the basioccipital recess, which separates into the two bulbous, expansive branches to join the rhomboidal recess (figure 14). Pharyngotympanic tubes connect to the rhomboidal recess ventrally, and the combined mediolateral width of the tubes is greater than their dorsoventral height (figure 14), as is also the case in other gavialoids [9,64]. Compared to other gavialoids, the pharyngotympanic sinus and intertympanic sinus of *Herugavialis* are more anteroposteriorly expansive, closer to the morphology seen in several crocodyloids [64,67]. The intertympanic sinus of *Herugavialis* possesses two parietal processes that extend anteriorly. Similar processes also characterize *Crocodylus*, *Gavialis* and *Thoracosaurus isorhynchus* [9,67,68]; however, those of the latter two taxa are larger and more dorsally expansive than those of *Herugavialis*.

**Figure 14.**
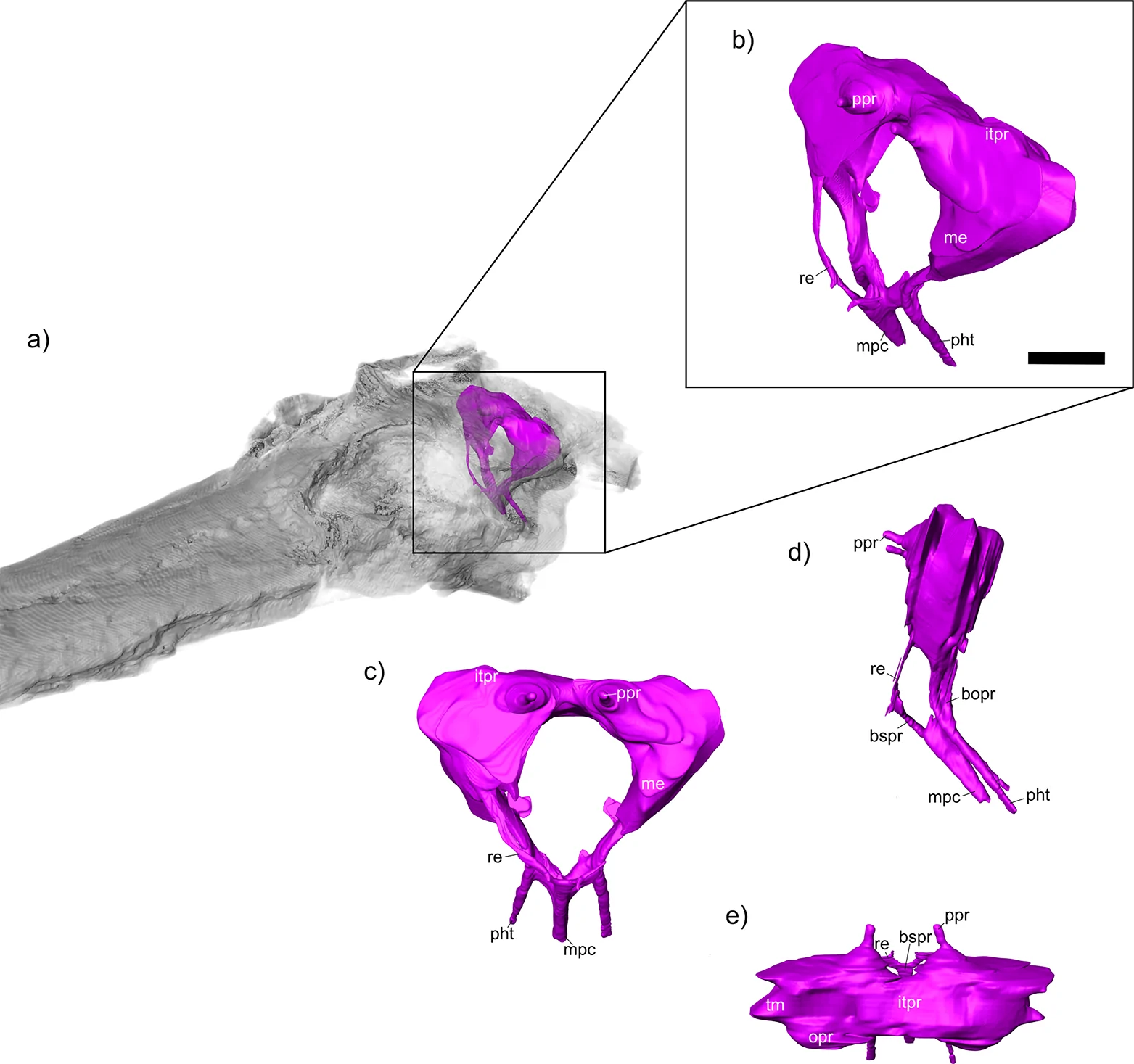
Paratympanic sinus system of the holotype skull (SMNS 10575) of *H. cairensis* n. gen. n. comb. (a) Skull rendering in left dorsolateral view, with the paratympanic sinus system in purple, (b) paratympanic sinus system in left dorsolateral view, (c) anterior view, (d) lateral view and (e) dorsal view. Abbreviations: bopr, basioccipital pneumatic recess; bspr, basiosphenoid pneumatic recess; itpr, intertympanic pneumatic recess; me, middle ear; mpc, median pharyngeal canal; opr, otoccipital pneumatic recess; pht, pharyngotympanic tubes; ppr, pre-parietal recess; re, *recessus epitubaricus*; tm, tympanic membrane. Scale bar,  10 mm.

### 4.25. Nasal cavity and associated structures

As the premaxilla is not preserved in SMNS 10575, the nasal cavity is only visible from the most anterior point of the maxilla, extending posteriorly towards the basicranium (figure 15). As in other gavialoids, the nasal cavity reflects the shape of the rostrum, expanding to form the olfactory region anterior to the orbits (figure 15). In the rostrum, the nasal cavity of *Herugavialis* is characterized by two channel-like ducts (neurovascular canals), which extend from the olfactory region anteriorly (figure 15).

**Figure 15.**
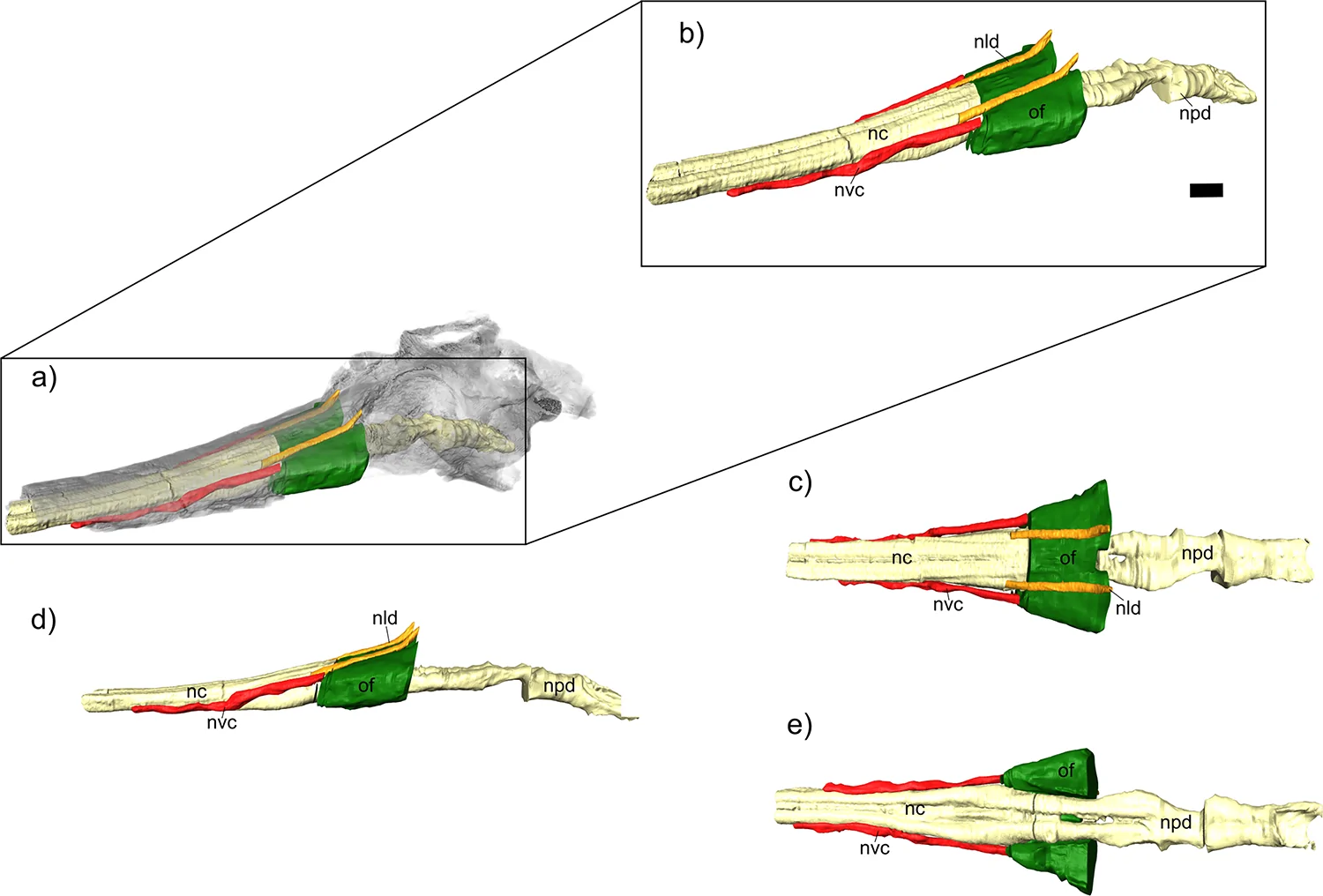
Nasal cavities of the holotype skull (SMNS 10575) of *H. cairensis* n. gen. n. comb. (a) Skull rendering in left dorsolateral view, with nasal cavities highlighted, (b) nasal cavities in left dorsolateral view, (c) nasal cavities in dorsal view, (d) nasal cavities in lateral view and (e) nasal cavities in ventral view. Abbreviations: nc, nasal cavity; nld, nasolacrimal duct; npd, nasopharyngeal duct; nvc, neurovascular canals; of, olfactory region. Scale bar, 10 mm.

The olfactory region is expansive and bulbous, resembling the condition in *E. lerichei* and marine thalattosuchian crocodylomorphs [9,69]. The nasopharyngeal ducts in *Herugavialis* are continuous with the nasal cavity and bifurcate into two ducts posterior to the olfactory region, ventral to the encephalic endocast (figure 15). They are bifurcated along their entire length and are more laterally expansive than in extant gavialoids, with a maximum mediolateral width comparable to that of the cerebrum (figure 15), similar to that of *E. lerichei* [9]. The olfactory region also protrudes over the nasopharyngeal ducts, as in *T. schlegelii* and *E. lerichei* (figure 15). In addition, the dorsal surface of the olfactory region in *Herugavialis* is characterized by two parallel nasolacrimal ducts that run anteroposteriorly (figure 15), which is most similar in morphology to those of *E. lerichei* and *T. schlegelii* [9]. The dorsal surface of the olfactory region of *Herugavialis* is expansive and bulbous, with the internal surface of the prefrontal and lacrimal bones correspondingly concave. This differs from the shallow depressions noted in many other gavialoid species (e.g. *Argochampsa krebsi*, *Eosuchus lerichei* and *Sutekhsuchus dowsoni*) [9,65], and most closely resembles the expansive depressions seen in the ‘thoracosaurs’ *Eothoracosaurus*, *Portugalosuchus* and *Thoracosaurus isorhynchus* [9,68].

## 5. New anatomical information on *Paratomistoma courti*

We note no major anatomical differences from the description of the external anatomy of the holotype skull of *P. courti* (CGM 42188) presented by Brochu & Gingerich [24], although we provide new, high-resolution photographs, alongside interpretative illustrations (figures 16–19).

**Figure 16.**
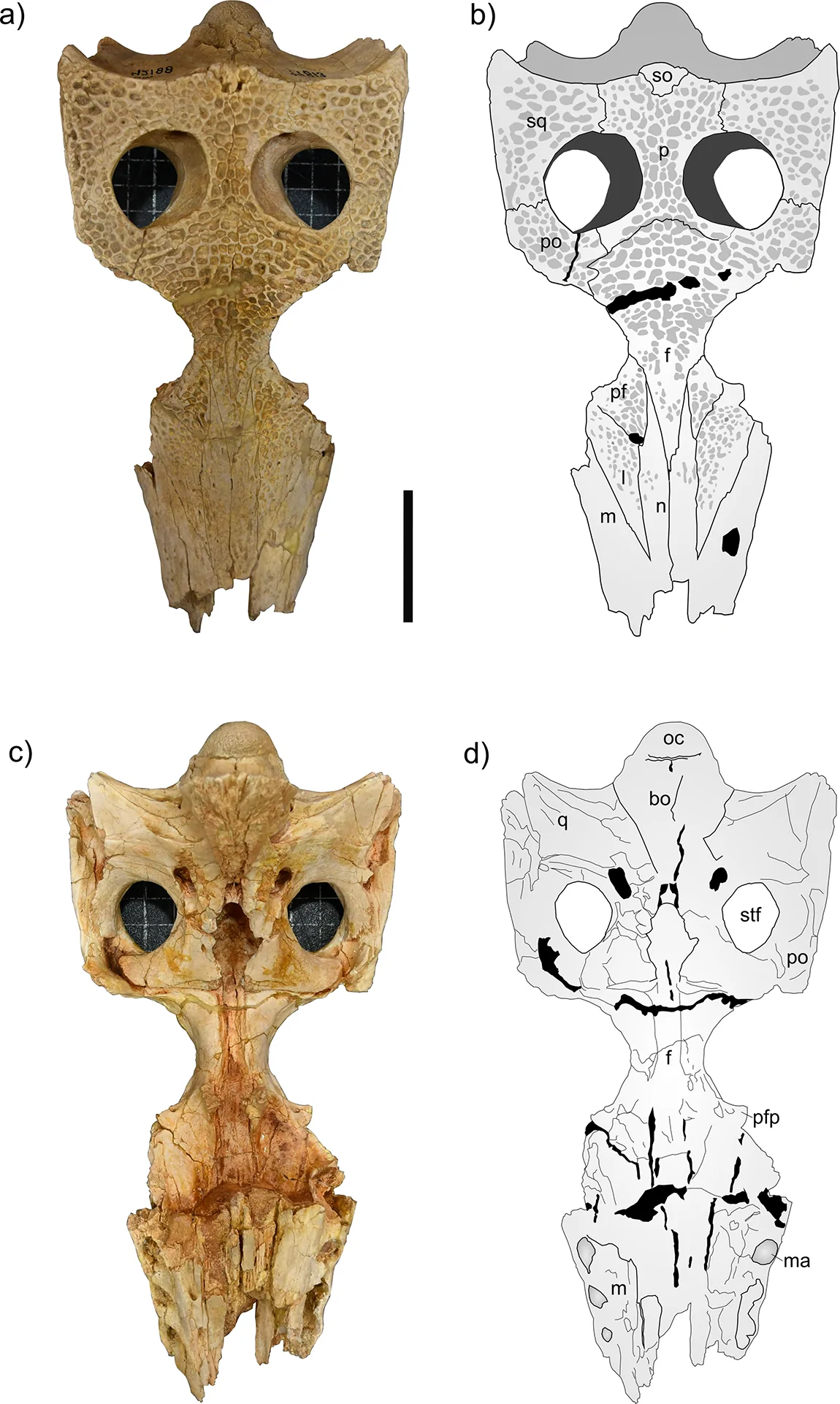
Holotype skull (CGM 42188) of *P. courti*. (a) Photograph of CGM 42188 in dorsal view, (b) interpretative drawing of CGM 42188 in dorsal view, (c) photograph of CGM 42188 in ventral view and (d) interpretative drawing of CGM 42188 in ventral view. Abbreviations. bo, basioccipital; f, frontal; l, lacrimal; m, maxilla; ma, maxillary alveoli; n, nasal; oc, occipital condyle; p, parietal; pf, prefrontal; po, postorbital; pfp, prefrontal pillar; q, quadrate; so, supraoccipital; sq, squamosal; stf, supratemporal fenestrae. Scale bar, 50 mm.

**Figure 17.**
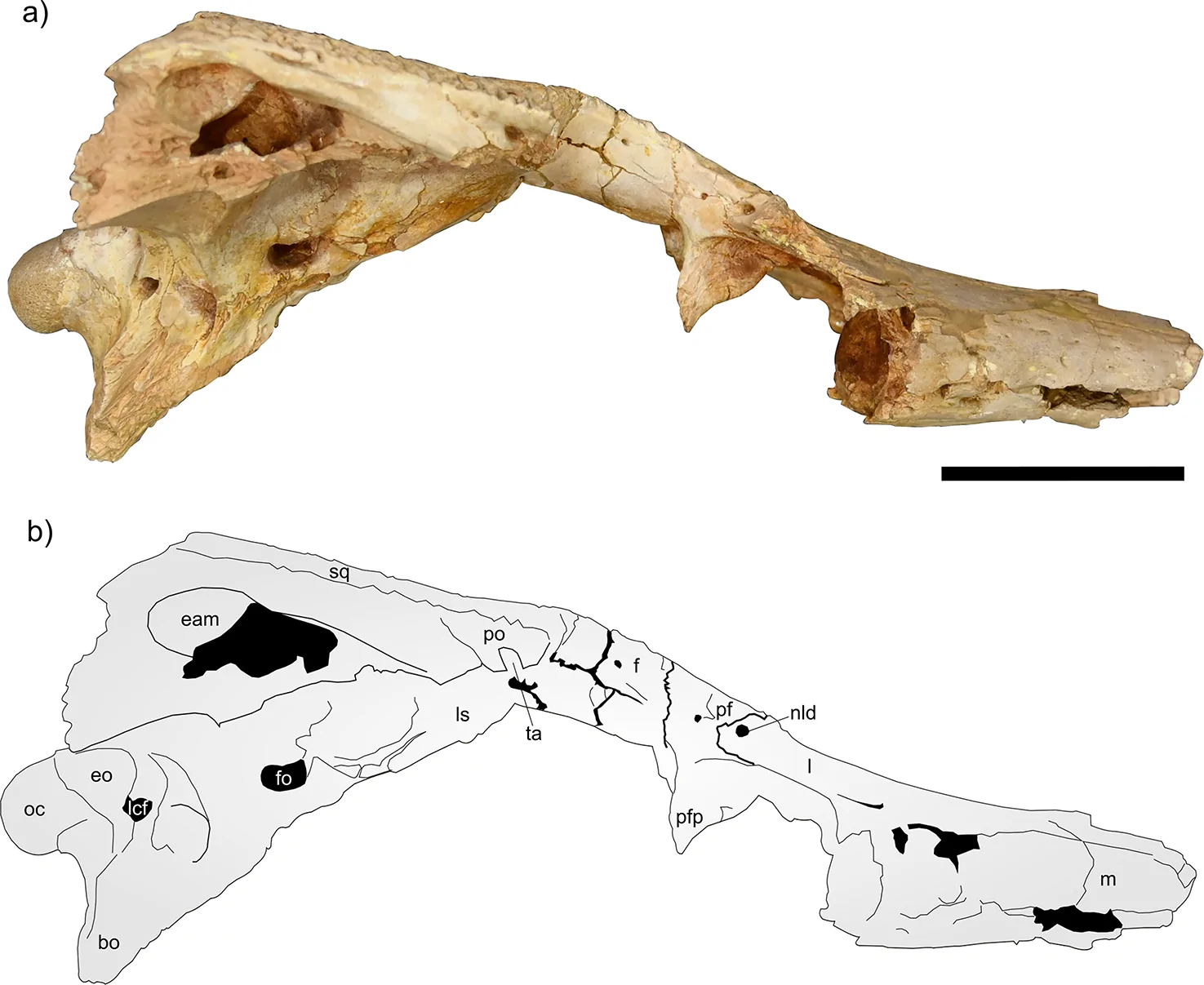
Holotype skull (CGM 42188) of *P. courti*. (a) Photograph of CGM 42188 in right lateral view and (b) interpretative drawing of CGM 42188 in right lateral view. Abbreviations. bo, basioccipital; eam, external auditory meatus; eo, exoccipital; f, frontal; fo, foramen ovale; l, lacrimal; ls, laterosphenoid; lcf, lateral carotid foramen; m, maxilla; nld, foramen for nasolacrimal duct; oc, occipital condyle; pf, prefrontal; pfp, prefrontal pillar; po, postorbital; sq, squamosal; ta, foramen for temporal artery. Scale bar, 50 mm.

**Figure 18.**
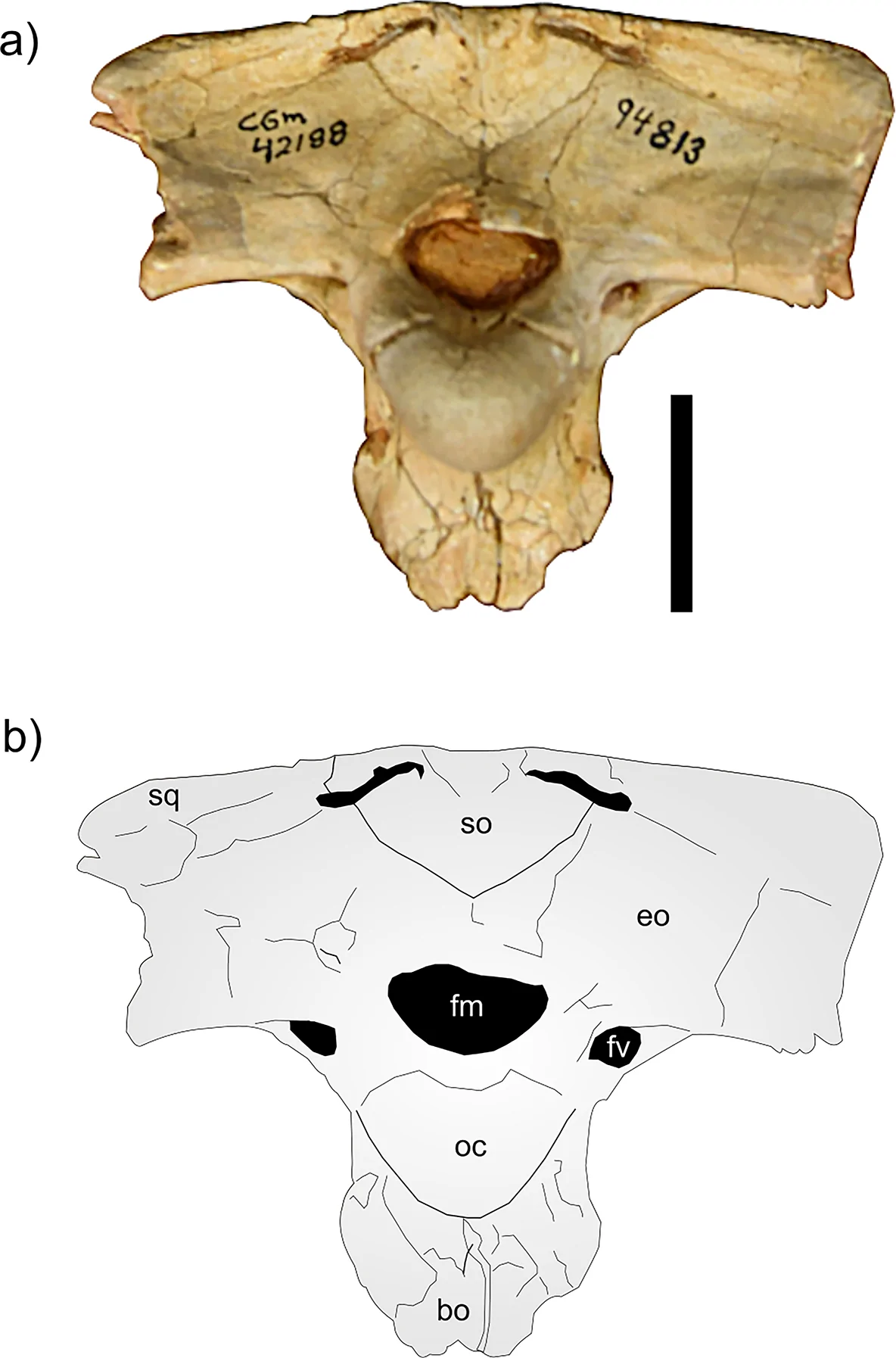
Holotype skull (CGM 42188) of *P. courti*. (a) Photograph of CGM 42188 in posterior view and (b) interpretative drawing of CGM 42188 in posterior view. Abbreviations. bo, basioccipital; eo, exoccipital; fm, foramen magnus; fv, vagus foramen; oc, occipital condyle; so, supraoccipital; sq, squamosal. Scale bar, 30 mm.

**Figure 19.**
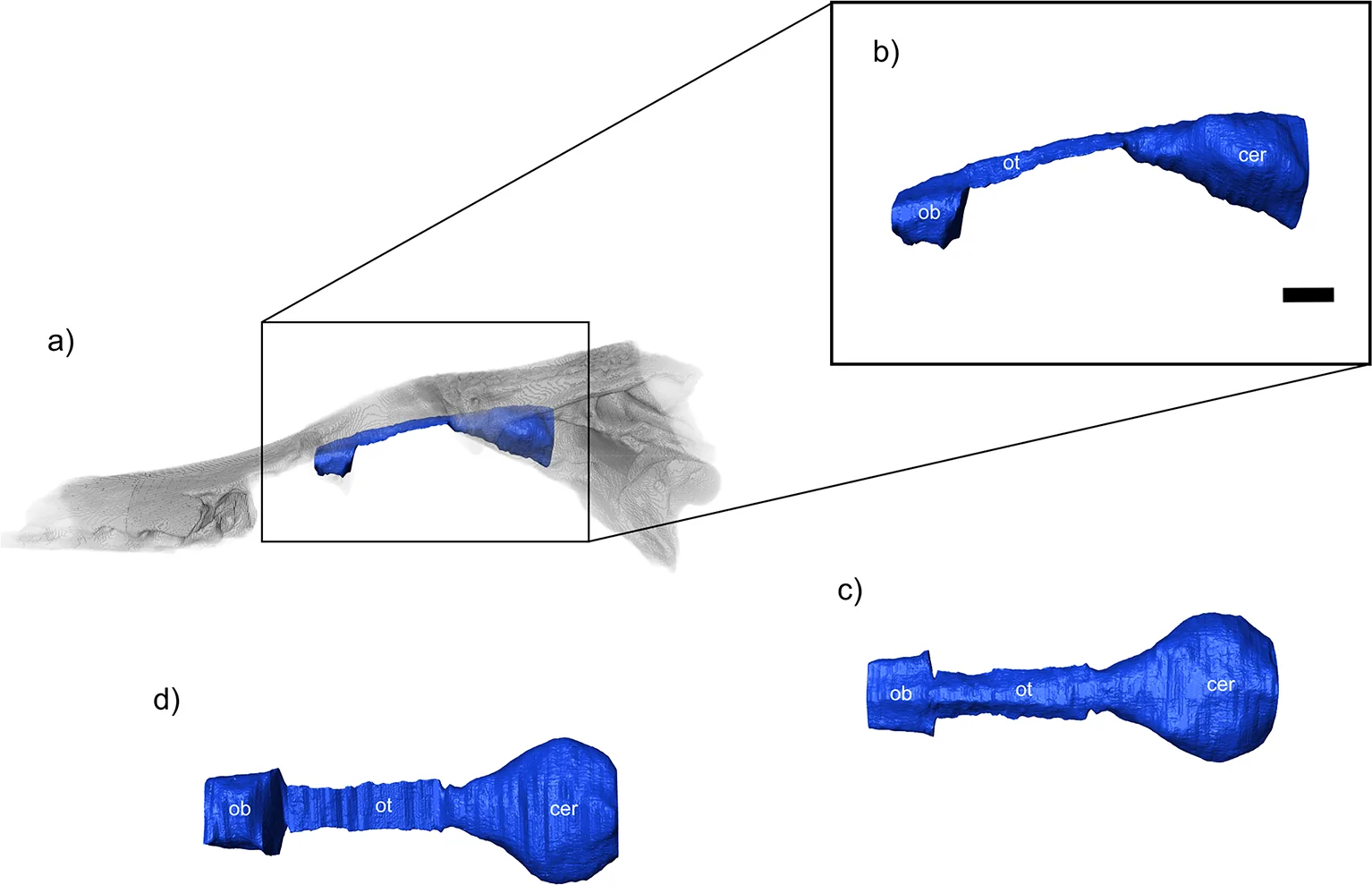
Encephalic endocast of the holotype skull (CGM 42188) of *P. courti*. (a) Skull rendering of CGM 42188 in left lateral view, with encephalic endocast in blue, (b) encephalic endocast in left lateral view, (c) dorsal view and (d) ventral view. Abbreviations: cer, cerebrum; ob, olfactory bulb; ot, olfactory tract. Scale bar, 10 mm.

The encephalic endocast of *P. courti* was partially segmented, with the olfactory bulb, tract and cerebrum preserved ( figure 19). As a result of the incomplete preservation, the cephalic and pontine flexure angles could not be estimated. The cerebrum of *Paratomistoma* has its greatest transverse width at its most posterior point, reducing in width anteriorly ( figure 19). The olfactory bulb is characterized by an increase in both mediolateral width and dorsoventral height from the olfactory tract ( figure 19). This morphology is most similar to that of *T. schlegelii*, contrasting with that of *G. gangeticus, Herugavialis* and other fossil gavialoids [9,63,65,68]. The anteroposterior length of the olfactory bulb of *Paratomistoma* is less than half that of the olfactory tract.

## 6. Phylogenetic results

Analysis with equal weighting resulted in 24 most parsimonious trees (MPTs) with a best score of 234 264.36 steps, a consistency index (CI) of 0.175 and a retention index (RI) of 0.654. The extended implied weighting analysis resulted in 9 MPTs, with a best score of 8567.48 steps, a CI of 0.171 and an RI of 0.647. The strict consensus trees of both analyses are depicted in figure 20. As with previous iterations of this matrix, the interrelationships of the major crocodylian clades remain unchanged, with Gavialoidea recovered as the sister group to Crocodyloidea, within Longirostres and Alligatoroidea outside this clade (e.g. [9,10,55,70,71]).

**Figure 20.**
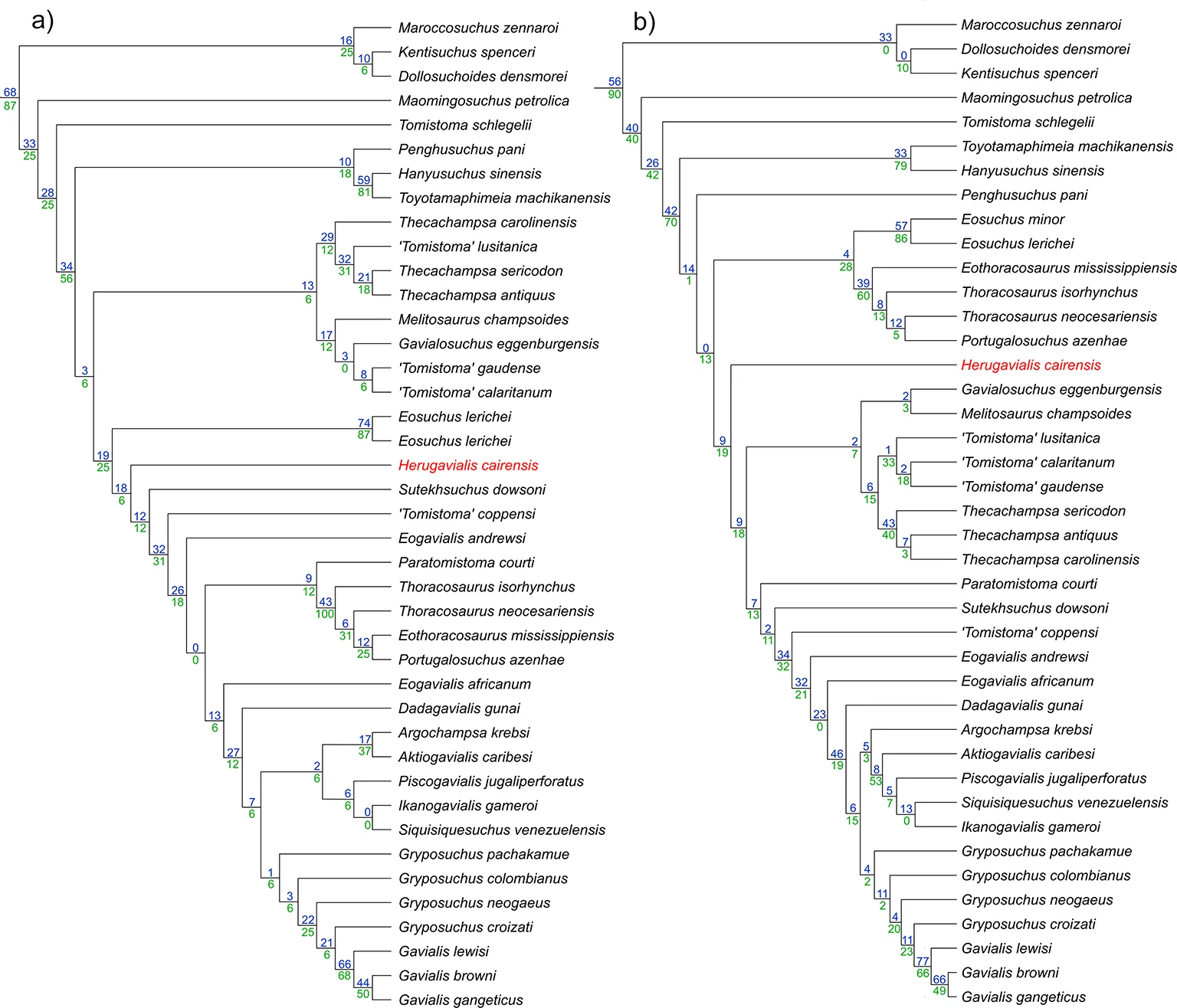
Tree topologies from (a) equal weighting and (b) extended implied weighting analyses. *H. cairensis* n. gen. n. comb is highlighted in red. Only the gavialoid part of the tree is shown. Bootstrap values are shown in blue; jackknife values are shown in green.

Within Gavialoidea, the interrelationships are mostly consistent with previous iterations of this matrix. Under both equal and extended implied weighting, the stem gavialoids, *Maroccosuchus*, *Dollosuchoides* and *Kentisuchus*, form the earliest diverging clade within Gavialoidea, with *Maomingosuchus* the sister taxon to Gavialidae ( figure 20). All gavialids are recovered as more closely related to *G. gangeticus* than to *T. schlegelii*. The East Asian taxa *Hanyusuchus, Penghusuchus* and *Toyotamaphimeia* are positioned as the earliest diverging gavialines ( figure 20). Broadly, the position of *Herugavialis* is consistent in that it is recovered as a relatively early diverging gavialine, outside the clade comprising *Gavialis* and gryposuchines. It also does not cluster with other African or Mediterranean taxa in either analysis ( figure 20). Under equal weighting, *Herugavialis* is part of a paraphyletic array of African taxa (also comprising *Sutekhsuchus*, ‘*Tomistoma*’ *coppensi* and *Eogavialis andrewsi*) outside a clade formed by ‘thoracosaurs’ (including *Paratomistoma*) and the *Gavialis* + gryposuchine clade ( figure 20a). *Eosuchus* is the sister taxon to the *Herugavialis* + *Gavialis* clade, with a large clade of European Miocene species (*Melitosaurus*, *Gavialosuchus* and several species currently assigned to ‘*Tomistoma*’) + *Thecachampsa* outside that grouping ( figure 20a). Under extended implied weighting, ‘thoracosaurs’ (now including *Eosuchus*) lie outside the *Herugavialis* + *Gavialis* clade ( figure 20b). The European Miocene species + *Thecachampsa* clade is now more phylogenetically nested than *Herugavialis*. Rather than a placement within ‘thoracosaurs’. *Paratomistoma* is instead recovered as part of the paraphyletic array of African species outside the clade comprising *Gavialis* and gryposuchines ( figure 20b).

Several synapomorphies support the phylogenetic placement of *Herugavialis* as a gavialine. Under equal weighting, this placement of *Herugavialis* is supported by (1) the maximum anteroposterior length of the cranial table being less than that of its maximum mediolateral width; (2) the absence of a posterior maxillary process that extends between the lacrimal and prefrontal; (3) the absence of a midsagittal crest on the frontal; (4) the absence of a supraoccipital process projecting from the midline of the cranial table; (5) the dorsal and ventral surfaces of the squamosal equally expanded laterally; (6) the foramen aereum positioned on the dorsomedial corner of the quadrate; (7) the exoccipital not reaching the articular border of the quadrate ramus; (8) the lateral carotid foramen separated from the metotic foramen; and (9) the absence of an ‘ear-shaped’ process projecting from the base of the prefrontal pillar.

Under extended implied weighting, the phylogenetic placement of *Herugavialis* as a gavialine is supported by the aforementioned synapomorphies 1, 6–9, in addition to (10) the minimum mediolateral width between the orbits being less than half that of the maximum mediolateral width across the anterior cranial table; (11) the absence of a supraoccipital process projecting from the midline of the cranial table; and (12) the dorsal and ventral margins of the squamosal not diverging anteriorly.

## 7. Discussion

### 7.1. Anatomical comparisons between *Herugavialis* and other Eocene Egyptian gavialoids

In addition to *H. cairensis* from the Lutetian (Lower Middle Eocene) Mokattam Formation, two further gavialoid species are currently recognized from the Eocene of Egypt: *P. courti* from the Bartonian (Upper Middle Eocene) Gehannam Formation [24] and *E. africanum* from the Priabonian (Upper Eocene) Qasr el-Sagha Formation to the lower Oligocene Gebel Qatrani Formation [47,72,73]. Here, we make anatomical comparisons between *Herugavialis* and these two species, a summary of which is listed in table 1.

**Table 1.** Summary of anatomical differences between the Eocene Egyptian gavialoids.

anatomical feature	*Herugavialis cairensis*	*Eogavialis africanum*	*Paratomistoma courti*
position of alveoli relative to maxillary palate	ventral	dorsal	?
position of maxilla–palatine suture on suborbital fenestra	anterior corner	anteromedial margin	?
position of anterior tip of frontal to that of the prefrontal	posterior	posterior	anterior
dorsomedial margin of orbit	strongly upturned and telescoped	raised from the skull surface	raised from the skull surface
dorsal profile of jugal	convex	sloping posteroventrally	?
ventrolateral sulcus at maxillary–jugal suture	absent	present	?
postorbital bar visible in dorsal view	visible	not visible	?
dorsal and ventral margins on the lateral surface of squamosal	parallel	diverge anteriorly	diverge anteriorly
ventral margin of squamosal projects farther laterally than dorsal margin on the lateral cranial table edge	absent	present	present
length of squamosal prong exceeds more than half the length of the cranial table	present	absent	?
orientation of the posterolateral surfaces of the skull table	dorsolateral	ventrolateral	ventrolateral
dorsal and ventral margins of the quadrate condyle	subparallel	constricted at mid-length	?
distance between paraoccipital process and quadrate condyle	greater than mediolateral width of condyle	less than mediolateral width of condyle	?
posterolateral tuberosities of the supraoccipital visible in dorsal view	absent	present	present
process of supraoccipital on dorsal surface of skull table	absent	present	absent
position of lateral carotid foramen to metotic foramen	ventral	lateral	ventral
posteroventral inclination of exoccipitals	present	present	absent
lateral margins of the basioccipital	parallel	diverge ventrally	parallel
dorsoventral height of the basioccipital greater than that of the occipital condyle	present	absent	absent
possession of sagittal crest on basioccipital	absent	present	absent
exposure of basisphenoid between basioccipital and pterygoids	little to no exposure	large, anteroposteriorly elongate exposure	?
extent of jugal relative to basioccipital tubera	extends beyond level of posterior margin	does not extend beyond posterior margin	?
posterior extent of mandibular symphysis reaches more than 13 alveoli	absent	present	?
anterior extent of splenial reaches more than 9 alveoli	absent	present	?

Contrasting with *Herugavialis*, in which the alveoli are positioned ventral to the maxillary palate, those of *E. africanum* are positioned dorsal to the maxillary palate [10]. Unlike *E. africanum*, where the maxilla–palatine suture is at the anteromedial margin of the suborbital fenestrae, the maxilla–palatine suture intersects the anterior corner of the suborbital fenestra in *Herugavialis* (figure 1) [10].

In both *Herugavialis* (figure 1) and *E. africanum* [47], the anterior tip of the frontal that is exposed on the skull surface is positioned posteriorly to the anterior tip of the prefrontal. By contrast, the anterior tip of the frontal of *Paratomistoma* is positioned anteriorly to that of the prefrontal [24]. Contrasting with both *E. africanum* and *Paratomistoma*, wherein the dorsomedial margin of the orbit is raised and not flush with the skull surface, the margins of the orbits in *Herugavialis* are strongly upturned and telescoped (figure 1).

The dorsal profile of the part of the jugal that forms the posteroventral margin of the orbit is convex and continuous with the dorsal margin of the lower temporal bar in *Herugavialis* (figure 2). In *E. africanum*, this dorsal profile of the jugal slopes posteroventrally, descending gradually into the lower temporal bar [10]. *Herugavialis* also lacks a ventrolateral sulcus at the maxillary–jugal suture, whereas this sulcus is present in *E. africanum* [10]. The jugal is not preserved in the holotype skull of *Paratomistoma*.

The postorbital bar in *E. africanum* is not visible in dorsal view [10]. This contrasts with the condition in *Herugavialis*, where the postorbital bar is laterally inclined, and thus visible in dorsal view (figure 1). In *P. courti* (figures 16 and 17), the postorbital bar is incomplete, with only its dorsalmost portion preserved [24].

In *Herugavialis*, the dorsal and ventral margins of the lateral surface of the squamosal are parallel to one another (figure 2), whereas they diverge anteriorly in both *E. africanum* and *Paratomistoma* (figure 17) [10]. In addition, the lateral cranial table edge is bevelled in the latter two taxa, wherein the ventral margin of the squamosal projects farther laterally than that of the dorsal margin [10,22]. In *Herugavialis*, the dorsal and ventral surfaces of the squamosal are equally expanded laterally (figure 2). *Herugavialis* is also characterized by long squamosal prongs, which exceed half the anteroposterior length of the cranial table, whereas the squamosal prongs are less than half the length of the cranial table in *E. africanum* [10,53,74,75]. The squamosal prongs are too incomplete in *Paratomistoma* to determine their extent (figure 16). The posterolateral surfaces of the skull table are orientated ventrolaterally from the sagittal axis in *Paratomistoma* and *E. africanum* [24,47]; however, these surfaces are orientated dorsolaterally in *Herugavialis* (figure 3).

Whereas the dorsal and ventral margins of the quadrate condyle are sub-parallel across their entire length in *Herugavialis* (figure 3), they are constricted at their mid-length in *E. africanum* [10,74]. The distance between the distal tip of the paroccipital process and the distal end of the quadrate condyle is greater than the mediolateral width of the quadrate condyle in *Herugavialis*, whereas it is less than that of the condyle width in *E. africanum* [10,22]. The quadrate condyle is not preserved in the holotype of *Paratomistoma*.

In dorsal view, the posterolateral tuberosities of the supraoccipital are not visible in *Herugavialis*, but they can be observed in both *E. africanum* and *Paratomistoma* (figures 1 and 16) [47]. Out of the three Eocene Egyptian gavialoids, *E. africanum* is the only gavialoid to possess a supraoccipital which possesses a process on the dorsal surface, wherein the lateral margins diverge from the main body of the supraoccipital at an angle greater than 10° [47,72].

Both *Herugavialis* and *Paratomistoma* are characterized by the lateral carotid foramen situated ventral to the metotic foramen, whereas it is positioned lateral to the latter foramen in *E. africanum*. The exoccipitals are posteroventrally inclined in *Herugavialis* and *E. africanum* such that they are visible in dorsal view (figure 5) [10], whereas they are subvertical in *Paratomistoma* (figure 16).

In both *Herugavialis* and *Paratomistoma*, the lateral margins of the basioccipital are parallel (figures 1 and 16), whereas they diverge ventrally in *E. africanum* [10]. *Herugavialis* differs from both *E. africanum* and *Paratomistoma* by possessing a basioccipital, wherein the dorsoventral height of the ventral plate is greater than that of the occipital condyle (figure 3). In the latter two species, the dorsoventral height of the ventral plate is less than that of the condyle [10,24]. Whereas *E. africanum* possesses a sagittal crest on the ventral plate of the basioccipital, this is absent in both *Herugavialis* and *Paratomistoma* [10].

In ventral view, there is little to no exposure of the basisphenoid between the basioccipital and pterygoids in *Herugavialis*. By contrast, *E. africanum* has a large, anteroposteriorly elongate basisphenoid exposure in this region [10]. In addition, the jugal extends beyond the level of the posterior margin of the basioccipital tubera in *Herugavialis* (figure 1), but does not extend beyond this margin in *E. africanum* [10].

The posterior extent of the mandibular symphysis only reaches the level of approximately 9–12 dentary alveoli in *Herugavialis* (figure 6), whereas it extends over 13 alveoli in *E. africanum* [10]. The anterior extent of the splenial in the dentary symphysis is adjacent to four to seven alveoli in *Herugavialis* (figure 6), but greater than seven alveoli in *E. africanum* [10].

In summary, there are numerous anatomical differences between *H. cairensis* and the other two Eocene Egyptian gavialoids, *P. courti* and *E. africanum*. Combined with their placement in phylogenetic analyses, wherein the three species are consistently recovered as distantly related to one another within Gavialoidea [9,10,12,13,17,23,25,76,this study], as well as their stratigraphic separation, this reinforces the hypothesis that they represent three distinct species. Consequently, given that *Herugavialis* also does not cluster with any other gavialoid taxon in our phylogenetic analyses, including *T. schlegelii*, this further supports our decision to erect a new genus name for ‘*Tomistoma*’ *cairense*.

### 7.2. Gavialoid diversity in the Mediterranean region

During much of the Cenozoic, the Mediterranean region hosted a high diversity of crocodylians, especially gavialoids. Unlike extant species, these extinct gavialoids frequented coastal and shallow marine habitats, with evidence for oceanic capabilities [6,7,17,24,65,77].

The Paleogene of the Mediterranean region was characterized by at least six gavialoid species (figure 21), namely *A. krebsi* from the Danian–Thanetian (Paleocene) Couche II and the Ypresian (Lower Eocene) Couche I levels of the Phosphate Series of Morocco [65,77], *Maroccosuchus zennaroi* from the Ypresian Couche I of Morocco [6,78], *H. cairensis* from the Lutetian (Lower Middle Eocene) Mokattam Formation of Egypt, *P. courti* from the Bartonian (Upper Middle Eocene) Gehannam Formation of Egypt [24], *Megadontosuchus arduini* from the Bartonian Orizzonte di Roncà Formation of Italy [3,79] and *E. africanum* from the Priabonian (Upper Eocene) Qasr el-Sagha Formation of Egypt [47,72].

**Figure 21.**
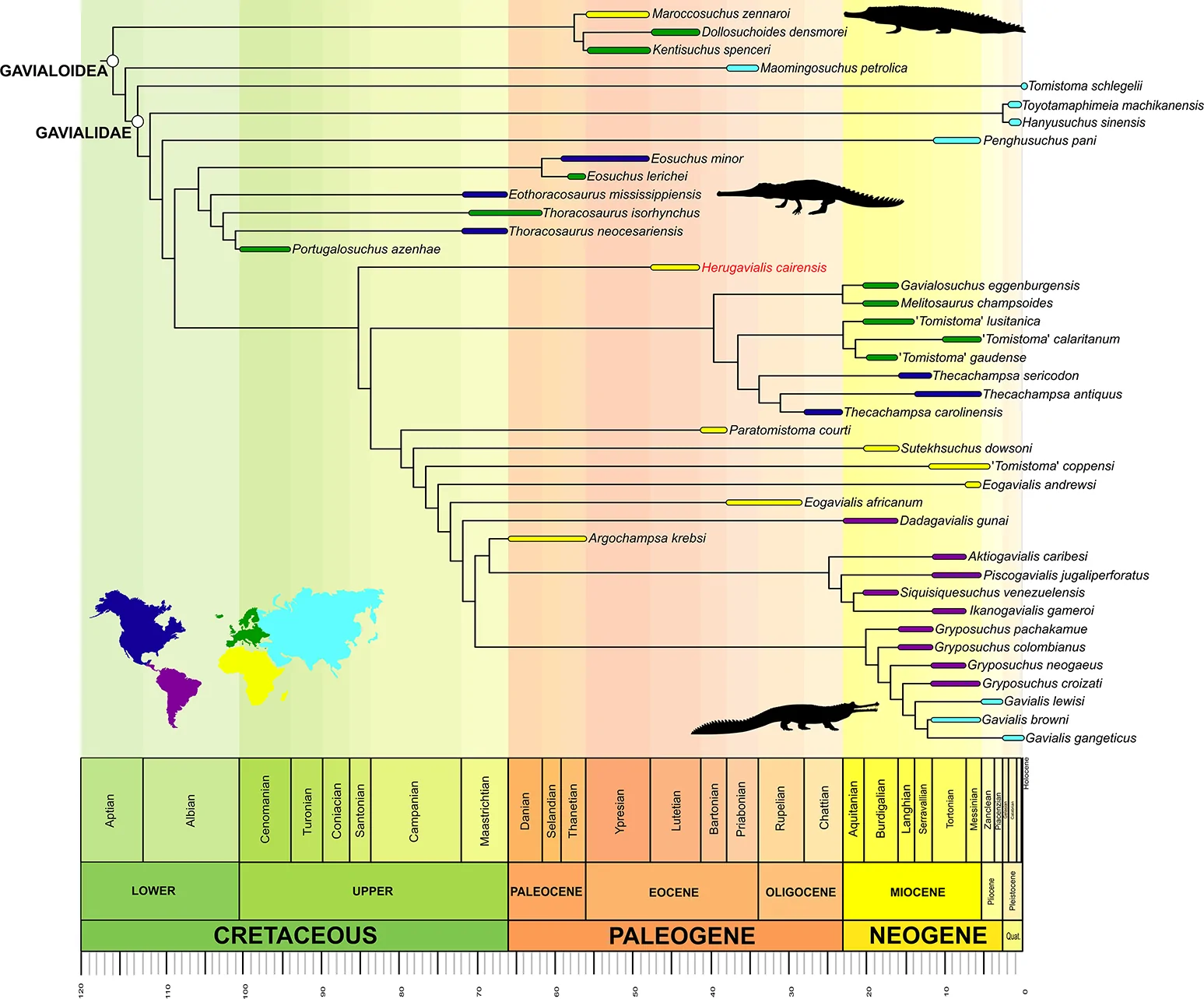
Time-scaled phylogeny of gavialoids based on results of extended implied weighting analysis. *H. cairensis* n. gen. n. comb is highlighted in red. Stratigraphic ranges taken from Rio & Mannion [10] and the Paleobiology Database (PBDB), and are colour-coded biogeographically: Africa = yellow, Asia = light blue; Europe = green; North America = dark blue; South America = purple. R package ‘strap’ [29] was used to time scale the tree.

The Miocene of the Mediterranean was also characterized by at least six gavialoid species (figure 21), namely *S. dowsoni* from the Burdigalian (Lower Miocene) Moghra Formation of Egypt and the Maradah Formation of Libya [17], *G. eggenburgensis* from the Burdigalian Bureschleinitz Formation of Austria [42], *Melitosaurus champsoides* and ‘*Tomistoma*’ *gaudense* from the Burdigalian Middle Globigerina Limestone Member of Malta [7,80,81], *‘Tomistoma’ lusitanica* from the Burdigalian of Portugal [82] and *‘Tomistoma’ calaritanum* from the Tortonian (Upper Miocene) Calcari di Cagliari Formation of Italy [7,83].

No gavialoid species are known from the Mediterranean region during the intervening interval (i.e. most or possibly all of the Oligocene). Although this might partly reflect sampling biases, it remains possible that this reflects a genuine signal, with global cooling during the Late Eocene–Oligocene lowering sea-surface temperatures and substantially reducing eustatic sea level [10,84,85], which might have reduced suitable habitat for gavialoids in the Mediterranean region [86]. Subsequent increases in eustatic sea level and temperature might therefore have facilitated the return to high gavialoid diversity in the Miocene. Support for a Late Paleogene Mediterranean extirpation of gavialoids, rather than a sampling failure-based explanation, comes from the results of our phylogenetic analyses, given that the Paleogene taxa do not cluster with the Miocene species (figure 21): if the latter were the descendants of the former, then we would expect them to form a clade. Currently, only the Miocene European gavialoids cluster together, forming a clade with the Oligocene–Miocene North American taxon *Thecachampsa* [10,17] (figure 21). The taxonomic classification of these European species awaits re-evaluation of ‘*Tomistoma*’ *lusitanica* and *G. eggenburgensis* (PMJB, unpublished data), which will facilitate greater insight into the evolutionary history of the Mediterranean gavialoids. Alongside other crocodylians in this region [87,88], Mediterranean gavialoids appear to have declined in the Late Miocene and had disappeared entirely prior to the Pliocene [7,20], with their extirpation possibly driven by the Messinian Salinity Crisis [89].

**Figure 22.**
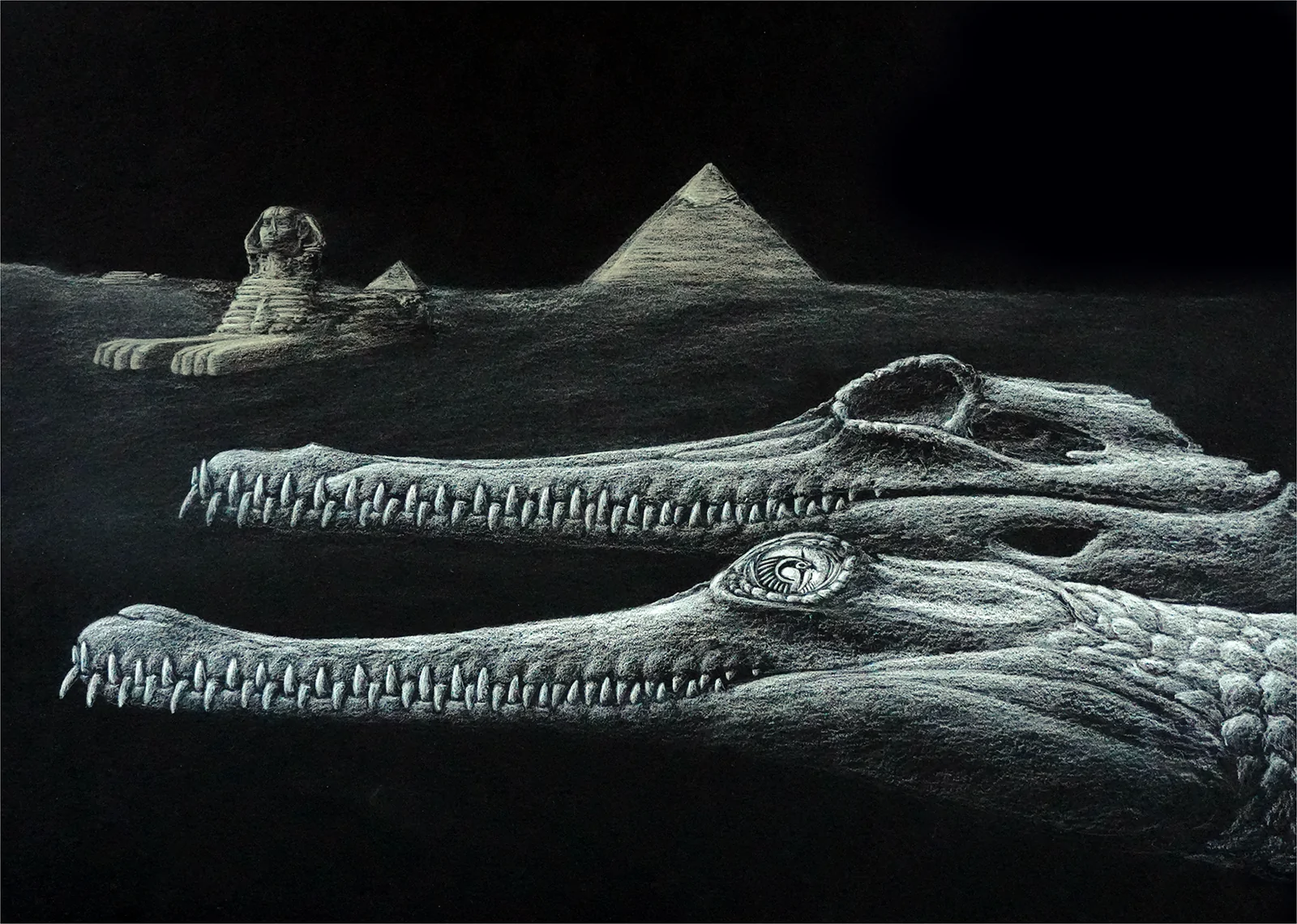
Life reconstruction of *Herugavialis cairensis* in left lateral view. *Herugavialis* is depicted with The Great Sphinx and pyramids of Giza in the background. Artwork by Karim Zanaty.

## 8. Conclusions

The redescription of the holotype and referred specimens of the Middle Eocene crocodylian species ‘*Tomistoma*’ *cairense* demonstrates numerous morphological differences from extant *T. schlegelii*. Coupled with the recognition of several autapomorphies, as well as its phylogenetic separation from *T. schlegelii*, we erect the new genus, *Herugavialis cairensis* (figure 22). *Herugavialis* can be differentiated from approximately contemporaneous Egyptian gavialoids by numerous anatomical features, exemplifying the diversity of gavialoids in the Mediterranean region during this period. Global cooling and a drop in eustatic sea level during the Late Eocene–Oligocene potentially limited suitable habitat for gavialoids in the Mediterranean region, with subsequent increases in eustatic sea level and temperature potentially facilitating the return to high gavialoid diversity observed in the Miocene. The taxonomic classification of some of these European species awaits re-evaluation, which will facilitate greater insight into the evolutionary history of the Mediterranean gavialoids.

## Supplementary Material



## Data Availability

All data can be found on Morphosource: https://www.morphosource.org/projects/000868536?locale%3Den. Supplementary material is available online [90].
