# Supplementary material for: Cranial anatomy of the Middle Eocene Egyptian crocodylian Herugavialis cairensis provides new insights into gavialoid diversity in the Mediterranean region
Source: R Soc Open Sci. 2026 Oct 7;13(10):261173. doi: 10.1098/rsos.261173 (PMC13641911; doi:10.1098/rsos.261173)
Supplement: Supplementary file 1 [file rsos.261173.zip › rsos.261173/rsos261173_si_001.docx]

Supplementary Materials for ’Cranial anatomy of the middle Eocene Egyptian crocodylian *Herugavialis cairensis* provides new insights into gavialoid diversity in the Mediterranean’

**Character score changes for *Herugavialis cairensis***

Character 9: 0 > ?

Character 12: 1 > ?

Character 15: 0 > ?

Character 54: ? > 0

Character 55: ? > 0

Character 56: ? > 0

Character 57: ? > 0

Character 58: ? > 0

Character 62: ? > 0

Character 63: ? > 0

Character 72: 1 > 2

Character 78: ? > 0

Character 93: 0 > ?

Character 95: 1 > 0

Character 102: 0 > ?

Character 115: ? > 0

Character 118: 1 > 0

Character 120: 0 > ?

Character 124: 0 > 1

Character 127: 0 > ?

Character 128: 0 > 1

Character 134: 0 > ?

Character 152: 0 > ?

Character 153: 0 > ?

Character 154: 0 > ?

Character 159: 0 > ?

Character 171: 1 > 0

Character 181: 0 > ?

Character 196: 0 > ?

Character 206: 0 > ?

Character 221: ? > 2

Character 228: 0 > ?

Character 230: 0 > ?

Character 232: 0 > ?

Character 234: 1 > ?

Character 239: 0 > ?

Character 240: 0 > ?

Character 244: 0 > ?

Character 245: 0 > ?

Character 246: 0 > ?

Character 247: 1 > ?

Character 249: 0 > ?

Character 250: 1 > ?

Character 251: 1 > ?

Character 252: 0 > ?

Character 253: 1 > ?

Character 254: 0 > ?

Character 256: 0 > ?

Character 331: ? > 0

**Character score changes for *Paratomistoma courti***

Character 89: 1 > ?

Character 105: 1 > ?

Character 108: ? > 1

Character 115: ? > 0

Character 128: 0 > 1

Character 130: 0 > 1

Character 132: 0 > 1

Character 151: 2 > ?

Character 238: 0 > ?

Character 256: 0 > ?
